# Gene networks driving bovine milk fat synthesis during the lactation cycle

**DOI:** 10.1186/1471-2164-9-366

**Published:** 2008-07-31

**Authors:** Massimo Bionaz, Juan J Loor

**Affiliations:** 1 Mammalian NutriPhysioGenomics, Department of Animal Sciences and Division of Nutritional Sciences, University of Illinois, Urbana, 61801 Illinois, USA

## Abstract

**Background:**

The molecular events associated with regulation of milk fat synthesis in the bovine mammary gland remain largely unknown. Our objective was to study mammary tissue mRNA expression via quantitative PCR of 45 genes associated with lipid synthesis (triacylglycerol and phospholipids) and secretion from the late pre-partum/non-lactating period through the end of subsequent lactation. mRNA expression was coupled with milk fatty acid (FA) composition and calculated indexes of FA desaturation and *de novo *synthesis by the mammary gland.

**Results:**

Marked up-regulation and/or % relative mRNA abundance during lactation were observed for genes associated with mammary FA uptake from blood (*LPL*, *CD36*), intracellular FA trafficking (*FABP3*), long-chain (*ACSL1*) and short-chain (*ACSS2*) intracellular FA activation, *de novo *FA synthesis (*ACACA*, *FASN*), desaturation (*SCD*, *FADS1*), triacylglycerol synthesis (*AGPAT6*, *GPAM*, *LPIN1*), lipid droplet formation (*BTN1A1*, *XDH*), ketone body utilization (*BDH1*), and transcription regulation (*INSIG1*, *PPARG*, *PPARGC1A*). Change in *SREBF1 *mRNA expression during lactation, thought to be central for milk fat synthesis regulation, was ≤2-fold in magnitude, while expression of *INSIG1*, which negatively regulates SREBP activation, was >12-fold and had a parallel pattern of expression to *PPARGC1A*. Genes involved in phospholipid synthesis had moderate up-regulation in expression and % relative mRNA abundance. The mRNA abundance and up-regulation in expression of *ABCG2 *during lactation was markedly high, suggesting a biological role of this gene in milk synthesis/secretion. Weak correlations were observed between both milk FA composition and desaturase indexes (i.e., apparent SCD activity) with mRNA expression pattern of genes measured.

**Conclusion:**

A network of genes participates in coordinating milk fat synthesis and secretion. Results challenge the proposal that *SREBF1 *is central for milk fat synthesis regulation and highlight a pivotal role for a concerted action among *PPARG*, *PPARGC1A*, and *INSIG1*. Expression of *SCD*, the most abundant gene measured, appears to be key during milk fat synthesis. The lack of correlation between gene expression and calculated desaturase indexes does not support their use to infer mRNA expression or enzyme activity (e.g., *SCD*). Longitudinal mRNA expression allowed development of transcriptional regulation networks and an updated model of milk fat synthesis regulation.

## Background

Progress in lactation biology of the bovine mammary gland advanced substantially during the 20^th ^century (review by [1]). Early studies with ruminants (1960 through 1980s) defined and quantified major metabolic aspects of mammary lipid metabolism, including *de novo *synthesis and fatty acid (**FA**) uptake from blood [2]. Milk lipid synthesis as well as droplet formation and secretion [3] received particular interest due to their influence on the manufacturing properties and organoleptic quality of milk and dairy products. Recent work has been more focused on qualitative aspects of lipid feeding to manipulate milk FA composition. Milk FA profiles and fat production are affected by stage of lactation and nutrition [4-6]. The latter, however, is by far the predominant environmental factor affecting milk fat production and it represents a practical tool to alter the yield and composition of FA regarded as functional food components (e.g., conjugated linoleic acid and omega-3 fatty acids; [1]). Clearly, deep understanding of mammary physiology and molecular adaptations to diet and/or physiological state are required for efficient manipulation of milk component synthesis and development of dairy products with specific characteristics (e.g., more unsaturated FA, more CLA).

Functional genomics studies highlighted the complexity and coordinated set of molecular events that encompass murine (reviewed in [7]), bovine [8], caprine [9], and porcine [10] mammary adaptations to lactation, revealing new insights about the underlying transcriptomic regulation [11]. Until recently bovine functional genomics studies were not feasible. However, up-to-date bovine genome sequencing and annotation efforts combined with quantitative PCR (**qPCR**) have become powerful tools for high-precision gene expression analysis. Genetic engineering studies in plants have revealed that an increase in metabolic flux requires manipulation of most of the enzymes in a biosynthetic pathway, challenging the idea of a "limiting enzyme" [12]. Therefore, measurement of mRNA for multiple genes and their networks in a pathway/s is essential to enable conclusions about a metabolic process and its outputs. Previous work in functional genomics also has reinforced the view that transcriptional regulation of gene expression is crucial because it is one of the major long-term regulatory mechanisms of cellular metabolism.

We recognize, however, that mRNA expression is one of multiple factors to be considered when studying the complex molecular networks working simultaneously in tissues. In fact, the ratio between mRNA abundance and abundance of the functional protein coded by the mRNA is hardly 1:1. This has been demonstrated in yeast, especially for the low abundant proteins [13]. There are numerous post-transcriptional and post-translational regulatory steps that preclude from inferring precisely protein abundance from mRNA. Numerous types of molecular and chemical relationships also exist which directly or indirectly (e.g., protein-protein interaction, phosphorilations) could affect protein activity. The fact remains that post-transcriptional regulation pertains more to short- than long-term regulation [14].

One of the long-term goals in our laboratory is to define gene networks involved in regulating mammary lipid synthesis in dairy cows. As an initial step to characterize these networks and their behavior, we have studied mammary tissue mRNA expression across changes in physiological state. Selected genes included those associated with FA uptake from blood, intracellular FA activation/channelling, *de novo *synthesis, desaturation, regulation of transcription, utilization of ketone bodies, phospholipid and triacylglycerol (**TAG**) synthesis, lipid globule membrane formation, as well as novel "lipogenic" genes (see Table 1 for details and gene description). Most of the selected genes were chosen based on previous studies with mammary tissue [2,6,15,16]. Others have only recently been discovered and their initial functional characterization conducted in mammary (e.g. *ABCG2 *[17]) or other tissues (e.g. *LPIN*, [18]). Specific isoforms for several families of genes involved in TAG synthesis were chosen based on previous published data from our laboratory [19]. The biological effect of changes in gene expression was evaluated via milk fatty acid secretion.

**Table 1:** Gene symbol, description, and overall % mRNA abundance among genes investigated

**FA import into cells**		**% RNA**^**1**^	**Triacylglycerol synthesis**		**% RNA**
*LPL*	Lipoprotein lipase	9.56	*GPAM*	Glycerol-3-phosphate acyltransferase, mitochondrial	2.31
*CD36*	CD36 molecule (thrombospondin receptor)	4.66	*AGPAT6*	1-acylglycerol-3-phosphate O-acyltransferase 6	1.28
*VLDLR*	Very-Low Density Lipoprotein Receptor	0.09	*DGAT1*	Diacylglycerol acyltransferase 1	0.14
**Xenobiotic and Cholesterol transport**			*DGAT2*	Diacylglycerol acyltransferase 2	<0.01
*ABCA1*	ATP-binding cassette, sub-family A (ABC1), member 1	0.07	*LPIN1*	Lipin 1	0.13
*ABCG2*	ATP-binding cassette, sub-family G (WHITE), member 2	8.54	**Regulation of transcription**		
**Acetate and FA activation and intra-cellular transport**			*INSIG1*	Insulin induced gene 1	0.35
*ACSS1*	acyl-CoA synthetase short-chain family member 1	0.33	*INSIG2*	Insulin induced gene 2	0.09
*ACSS2*	acyl-CoA synthetase short-chain family member 2	0.59	*SCAP*	SREBP cleavage activating protein	0.13
*ACSL1*	Acyl-CoA synthetase long-chain family member 1	0.89	*SREBF1*	Sterol regulatory element-binding transcription factor 1	0.15
*ACBP*	Acyl-CoA binding protein (diazepam binding inhibitor)	0.17	*SREBF2*	Sterol regulatory element-binding transcription factor 2	0.10
*FABP3*	Fatty acid-binding protein, heart	15.49	*THRSP*	Thyroid hormone responsive SPOT14	0.01
**Fatty acid synthesis and desaturation**			*PPARG*	Peroxisome proliferator-activated receptor gamma	0.01
*ACACA*	Acetyl-coenzyme A carboxylase alpha	0.91	*PPARGC1A*	PPAR gamma, coactivator 1 alpha	0.04
*FADS1*	Fatty acid desaturase 1 (delta-5 desaturase)	0.20	*PPARGC1B*	PPAR gamma, coactivator 1 beta	0.01
*FADS2*	Fatty acid desaturase 2 (delta-6 desaturase)	<0.01	**Sphingolipid synthesis**		
*FASN*	Fatty acid synthase	7.05	*SPTLC1*	Serine palmitoyltransferase, long chain base subunit 1	0.15
*SCD*	Stearoyl-CoA desaturase (delta-9-desaturase)	23.14	*SPTLC2*	Serine palmitoyltransferase, long chain base subunit 2	0.15
**Lipid droplet formation**			*LASS2*	LAG1 homolog, ceramide synthase 2	0.61
*ADFP*	Adipose differentiation related protein (adipophilin)	9.56	*SPHK2*	Sphingosine kinase 2	0.09
*BTN1A1*	Butyrophilin, subfamily 1, member A1	4.78	*ASAHL*	N-acylsphingosine amidohydrolase-like	0.05
*XDH*	Xanthine dehydrogenase	7.39	*SGPL1*	Sphingosine-1-phosphate lyase	0.06
*PLIN*	Perilipin	0.01	*UGCG*	Ceramide glucosyltransferase	0.18
**Ketone body utilization**			*OSBP*	Oxysterol-binding protein 1	0.12
*BDH1*	3-hydroxybutyrate dehydrogenase, type 1	0.02	*OSBPL2*	Oxysterol binding protein-like 2	0.17
*OXCT1*	3-oxoacid CoA transferase 1	0.07	*OSBPL10*	Oxysterol binding protein-like 10	0.06

^1 ^The % mRNA abundance is calculated by [((1/E^ΔCt^) specific gene/sum (1/E^ΔCt^) all genes) × 100]. See Materials and Methods for details

## Methods

### Animals, sampling, and diet

Holstein dairy cows of high genetic merit were used (Additional file 1, Table S1). Details of the experimental design were reported previously [20]. Briefly, percutaneous biopsies from each of 6 cows were obtained from the right or left rear quarter of the mammary gland at -15 (-13 ± 3), 1, 15, 30, 60, 120, and 240 d relative to parturition.

### RNA extraction, PCR, and design and evaluation of primers

Specific details of these procedures are presented in the Additional file 1 (Supplementary Materials and Methods and Table S2, Table S3, and Table S4).

### Data processing and statistical analysis

PCR-normalized data are presented as *n*-fold change relative to -15 d. To estimate standard errors at -15 d, and prevent biases in statistical analysis, normalized data were transformed to obtain a perfect average of 1.0 at -15 d, leaving the proportional difference between the biological replicates. The same proportional change was calculated at all other time points to obtain a fold change relative to -15 d. This final dataset was analyzed using a MIXED model with repeated measures in SAS (SAS Inst. Inc. Cary, NC, release 8.0) to evaluate the effect of time relative to parturition on gene expression. Compound symmetry was the most appropriate covariate structure used for repeated measures analysis. The model included the fixed effect of time (-15, 1, 15, 30, 60, 120, and 240 d) and the random effect of cow.

### Relative mRNA abundance among transcripts

Efficiency of PCR amplification for each gene was calculated using the standard curve method (E = 10^-1/-log curve slope^) (Additional file 1, Table S5). Relative mRNA abundance among measured genes was calculated as previously reported [19], using the inverse of PCR efficiency raised to ΔCt (gene abundance = 1/E^ΔCt^, where ΔCt = Ct sample - geometric mean Ct of 3 internal control genes). Overall mRNA abundance for each gene among all samples measured was calculated using the median ΔCt. Use of this technique for estimating relative mRNA abundance among genes was necessary because relative mRNA quantification was performed using a standard curve (made from a mixture of RNA from several bovine tissues [20]), which precluded a direct comparison among genes. Together, use of Ct values corrected for the efficiency of amplification plus internal control genes as baseline overcome this limitation. Description of genes measured and overall % relative mRNA abundance are reported in Table 1.

### Milk yield, composition and fatty acid analysis

Specific details regarding measurement of milk yield, composition, and fatty acid analysis are presented in the Additional file 1. Daily yield of fatty acids (mole/d) synthesized *de novo *was calculated by the sum of FA 4-14-carbon FA, and yield of FA taken up from blood by the sum of 18-24-carbon FA. The index of acetyl-CoA incorporated during FA elongation (**ACE **or FA synthesis from acetyl-CoA) was calculated as suggested previously [5] with modifications (see caption in Additional file 1, Table S6 for details).

### Gene network analysis

Gene networks were evaluated using Ingenuity Pathway Analysis^® ^(IPA; , Redwood City, CA). This is a web-based application that enables the discovery, visualization, and exploration of interaction networks. The software relies on currently known relationships (i.e., published manuscripts) among human, mouse, and rat genes/proteins.

## Results and Discussion

### Milk fatty acid composition: a functional analysis

**Figure 1:**
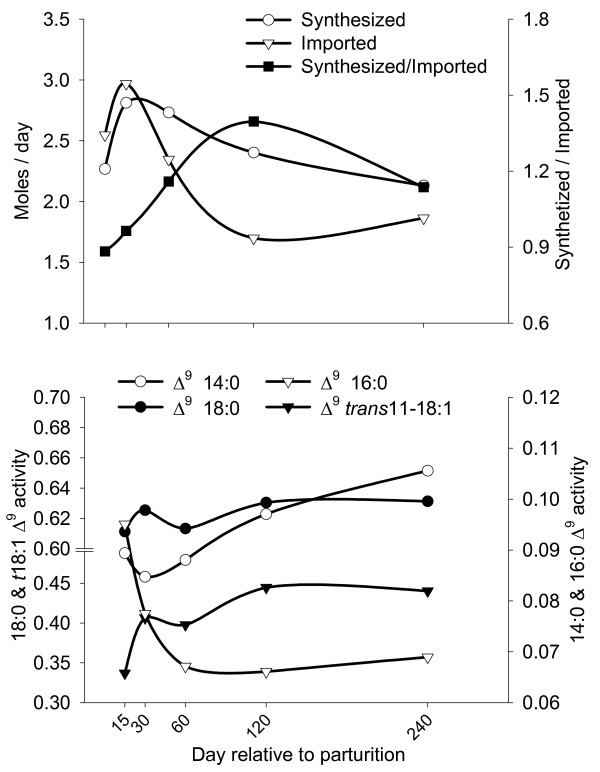
***De novo *vs. imported FA and Δ^9 ^desaturase indexes**. *De novo *FA synthesis (Synthesized) *vs *FA import (Imported) analysis (Top panel) and Δ^9 ^desaturase indexes during lactation (Bottom panel). Synthesized = FA with 4÷14 carbons except 11:1; pooled SEM = 0.34. Imported from blood = FA with carbon chain >16 plus 15:0 and 11:1; pooled SEM = 0.31. Synthesized/Imported, pooled SEM = 0.10. Pooled SEM for Δ^9 ^activity on 14:0, 16:0, 18:0, and *trans*11-18:1 was 0.008, 0.007, 0.02, and 0.06, respectively. Statistical effect of time: *P *< 0.05 for all measurements except Synthesized FA (*P = *0.24) and Δ^9 ^activity on 18:0 (*P *= 0.77).

Lactation patterns of FA synthesized vs. FA taken up (Figure 1, top panel) suggest that uptake from blood predominated during the first mo of lactation. Calculation of synthesized FA (Figure 1, top panel) and ACE data (Table 2) suggest that synthesis of FA from acetate or butyrate began during the first 2 wk of lactation and increased rapidly thereafter reaching a peak at 30 d. Thus, we concluded based also on ratio of synthesized/imported (Figure 1, top panel, Table 2) that *de novo *FA synthesis predominated after 1^st ^mo post-partum. The simple sum of FA yield (Σ4- to 14-carbon FA) to estimate amount of FA originating *de novo *suffers from the assumption that butyrate in milk is completely derived from *de novo *synthesis. Reports indicated that the major part of butyrate (derived from β-hydroxybutyrate) is incorporated directly into *de novo*-synthesized FA (50–60%) [16]. However, a considerable portion also is esterified directly into the *sn-*3 of TAG [2,21]. Pattern of ACE (Table 2) corresponded with pattern of *ACACA *(acetyl-coenzyme A carboxylase alpha) and *FASN *(fatty acid synthase) expression (peaked at 60 d, see "Concerted action between *de novo* FA synthesis and desaturation in mammary TAG synthesis" section for details), both of which are key enzymes regulating *de novo *synthesis. Additional discussion on milk FA composition is available in Additional file 1 (Supplementary Results and Discussion).

**Table 2:** % FA from blood, synthesized *de novo*, and calculated incorporation of acetyl-CoA into *de novo *synthesized FA (ACE)^1^

	Day relative to parturition						
			
Item	15	30	60	120	240	SEM	*P*-Value^2^
Synthesized FA %^3^	46.4^a^	48.9^ab^	53.5^bc^	57.8^c^	52.8^abc^	2.08	< 0.01
FA from blood %^4^	53.6^c^	51.1^bc^	46.5^ab^	42.2^a^	47.2^abc^	2.08	< 0.01
ACE mol/d^5^	5.2^a^	14.0^b^	13.8^b^	12.9^b^	12.0^b^	1.46	< 0.01
ACE mol/d^6^	4.9^a^	13.7^b^	13.5^b^	12.6^b^	11.7^b^	1.42	< 0.01
Δ^9^-Desaturase index^7^	0.35^b^	0.33^b^	0.30^ab^	0.27^a^	0.30^ab^	0.17	< 0.01

^1 ^The complete FA datasets (mole/day and g/100 g) are reported in Additional file 1 (Tables S6 and S7).

^2^Effect of day.

^3 ^Fatty acid with carbon length 4÷14 (except 11)/tot FA (except 16:0 and 16:1).

^4 ^Fatty acid with carbon length >16 (included 11 and 15)/tot FA (except 16:0 and 16:1).

^5 ^ACE corrected (Additional file 1, Table S6).

^6^ACE corrected without considering odd chain FA (11:0, 15:0) (Additional file 1, Table S6).

^7 ^Overall Δ^9 ^desaturase index, calculated from (14:1 *c*9 + 16:1 *c*9 + 18:1 *c*9 + 18:2 *c*9, *t*11)/(14:0 + 14:1 *c*9 + 16:0 + 16:1 *c*9 + 18:0 + 18:1 *c*9 + 18:1 *t*11 + 18:1 *c*9, *t*11)

^a,b,c ^denote *P *< 0.05

### Fatty acid uptake by mammary cells

#### LPL and VLDLR and exogenous FA utilization

**Figure 2:**
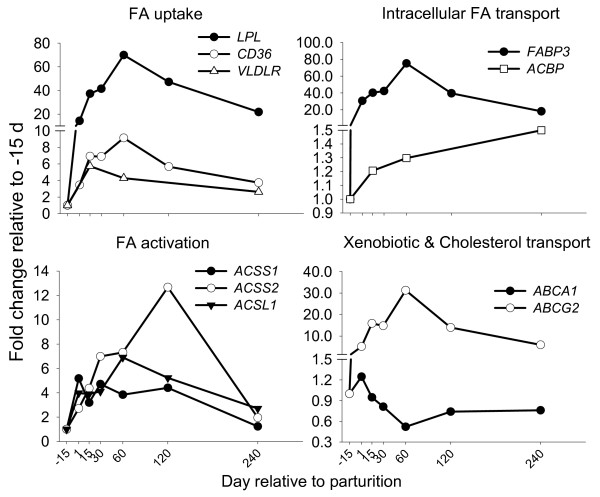
**Genes involved in FA uptake, activation, intracellular trafficking, and xenobiotic and cholesterol transport**. Temporal expression patterns in bovine mammary of genes involved in FA uptake (*LPL*, SEM = 8.0; *CD36*, SE = 0.97; *VLDR*; SEM = 0.72), FA and acyl-CoA transport (*FABP3*, SEM = 6.18; *ACBP*, SE = 0.11), short- and long-chain FA activation (*ACSS1*, SEM = 0.95; *ACSS2*, SEM = 1.66; *ACSL1*, SEM = 0.61), and xenobiotic and cholesterol transport (*ABCA1*, SEM = 0.22; *ABCG2*, SEM = 2.69). Statistical effect of time: *P *< 0.05 for all genes except *ABCA1 *(*P *= 0.06).

Mammary cells take up LCFA from albumin-bound fatty acids (NEFA) and lipoproteins. VLDL or chylomicrons are anchored to mammary endothelium by lipoprotein lipase (LPL), which then hydrolyzes TAG in the lipoprotein core to release FA [22]. LPL has higher activity in mammary [22] compared with other tissues, probably due to its high mRNA abundance. The observed up-regulation in *LPL *mRNA as early as the onset of milk synthesis was remarkable (Figure 2) because mouse mammary tissue had only a 2-fold increase in *LPL *transcript between pregnancy and lactation. In the mouse, the increase was accompanied by 2-fold up-regulation of enzymatic activity [23,24]. In contrast with murine, bovine mammary *LPL *expression pattern was remarkably similar to the lactation curve (Additional file 1, Figure S1), which might be indicative of an important role of this gene in maintenance of milk synthesis.

Recent evidence points at VLDL receptor (VLDLR) as an essential component of LPL activity [25]. *VLDLR *expression was up-regulated throughout lactation, particularly in the first mo post-partum (Figure 2). The mRNA abundance of *LPL *and *VLDLR *accounted for ~10% and ~0.1% of total genes measured (Table 1). Despite these differences, data suggest an important role for VLDLR in concert with LPL [25] in milk fat synthesis during lactation. Mammary VLDLR could act on chylomicrons or intestinal VLDL, which contain apo-B48 [26]. In general, our data are in agreement with previous work reporting higher efficiency of mammary TAG uptake from lipoproteins at the beginning of lactation [26]. The pattern of mammary tissue expression of *LPL *during lactation was in accordance with the typical increase in blood LDL in dairy cows post-partum, which is an indirect index of VLDL utilization [27].

#### FAT/CD36 and FA internalization

Passive diffusion of FA across membranes plays a minor role compared with protein-mediated FA uptake and the flip-flop mechanism [28]. The main proteins involved in FA uptake in non-ruminant cells include fatty acid translocator FAT/CD36 (*CD36*) and fatty acid transport proteins (FATP or *SLC27A*) [28]. *CD36 *mRNA in our study accounted for ~5% of total genes measured (Table 1) and had a large increase in expression (>8-fold) during lactation (Figure 2). This protein is believed to participate in the process of milk fat secretion [3] because of its presence in the milk fat globule membranes (**MGFM**) [29]. Our data support an important role for CD36 in milk fat synthesis. Although a role for this gene in milk fat secretion cannot be excluded we believe that its involvement in FA import in bovine mammary cells is more important.

We previously showed that bovine mammary tissue expresses most of the known SLC27A isoforms, but only expression of *SLC27A6 *was up-regulated during the first mo of lactation suggesting a role in NEFA uptake [19]. Up-regulation in expression of *SLC27A6 *and *CD36*, and the fact that their proteins co-localize in murine heart subcellular fractions, support the concept of cooperation between both proteins during FA uptake. CD36 also co-localizes with acyl-CoA synthetases (ACSL) and fatty acid binding proteins (FABP) [30]. Clearly, FA uptake by bovine mammary cells is a complex and coordinated mechanism requiring evaluation of multiple genes/proteins.

### Activation and intracellular channelling of FA

#### ACSL1 and ACSS2 and FA activation for milk TAG

Long-chain FA (**LCFA**) are esterified with CoA in the inner face of the plasma membrane prior to participating in metabolic pathways. FA activation occurs primarily via acyl-CoA synthetase long-chain family member isoforms (ACSL) [31]. *ACSL1 *mRNA is predominant among *ACSL *isoforms in bovine mammary tissue [19], and it increased >4-fold at the onset of lactation suggesting this isoform is important for copious milk fat synthesis (Figure 2). Among enzymes involved in activation of short chain FA (**SCFA**), acyl-CoA synthetase short-chain family member 2 (*ACSS2*) had greater mRNA abundance and up-regulation in expression than *ACSS1 *(a.k.a. *ACAS2L*). mRNA abundance of *ACSS1*, *ACSS2*, and *ACSL1 *in each case was <1% of genes investigated (Table 1). Bovine ACSS isoforms have been isolated and characterized in tissues other than mammary [32]. In the mouse, ACSS isoforms only have 43.8% amino acid similarity and are located in different cell compartments. ACSS2 (originally named AceCS1) is exclusively present in cytosol, while ACSS1 (originally named AceCS2) is primarily found in mitochondria [32]. Both enzymes have high affinity for acetate, with ACSS2 showing greater affinity than ACSS1. The latter also has modest affinity for propionate [32].

Bovine ACSS1 activated >4-fold more ^14^C-acetate into CO_2 _than lipid, suggesting it targets acetate towards oxidation [32]. Human ACSS2 was shown to channel acetate towards FA synthesis [33]. In our study, both *ACSS1 *and *ACSS2 *mRNA increased substantially during lactation (Figure 2). *ACSS2 *transcript pattern corresponded with bovine mammary acetyl-CoA production throughout lactation [34]. Thus, its large increase at the onset of lactation along with the pattern of ACE during the first 60 d post-partum, suggest the protein encoded by this gene provides activated acetate for *de novo *FA synthesis. In addition to its use in FA synthesis, acetate is the chief carbon source for energy generation in mammary accounting for ~33% of total CO_2 _produced by the tissue [35]. Lower mRNA abundance and pattern of expression of *ACSS1 *throughout lactation is in agreement with acetate use for oxidation [2]. Overall, *ACSS *isoforms expression reflected the need for activation of acetate in mammary tissue.

#### FABP3 and FA trafficking towards milk TAG

Free diffusion of LCFA into cells is too slow to account for the rapid transport and selective targeting towards specific organelles [36], thus, LCFA require specific transporters. Fatty acid binding protein (FABP) and acyl-CoA binding protein (ACBP or DBI) are the main intracellular FA transporters in non-ruminant cells [36]. The former has high affinity for LCFA but also can bind acyl-CoA [37,38]. ACBP is the major intracellular transporter of acyl-CoA in several mammalian tissues [39]. We previously observed the presence of mRNA of all *FABP *isoforms, except *FABP2 *mRNA, in bovine mammary tissue with greater abundance and up-regulation of *FABP3 *mRNA during lactation. Transcript of *FABP4 *and *FABP5 *also were up-regulated during lactation but were less abundant compared with *FABP3 *[19]. In the present study, *FABP3 *was the second most abundant transcript (~16%) among all measured, in accord with the large cytosolic content of its protein in mammary epithelium [38]. The large mRNA abundance of this gene also was a consequence of the 80-fold up-regulation during lactation, whereas *ACBP *mRNA abundance was <0.2% among all genes and had a small increase (1.5-fold) during lactation (Table 1 and Figure 2). Low *ACBP *mRNA abundance agrees with protein abundance data in bovine mammary [40]. Our results suggest a minor role of *ACBP *in bovine mammary lipid synthesis, also supported by murine data [23].

In addition to a trafficking role, FABP3 through binding of activated acyl-CoAs could buffer cells from negative effects of activated FA and prevent inhibition of ACACA and SCD (stearoyl-CoA desaturase), roles usually attributed to ACBP [39]. A positive relationship between FABP and SCD has been demonstrated in chickens [41], indicating a coordinated function between both proteins in mammary tissue as also suggested previously from an evaluation of published data [6]. Based on our longitudinal mRNA expression and fatty acid data we propose that an important function of FABP3 in bovine mammary is to provide FA for SCD. Large affinity of FABP4 for oleic acid and up-regulation of its mRNA during lactation in bovine mammary tissue [19], led us to propose that FABP3 provides stearoyl-CoA (or other substrates such as 16:0 and *trans*11-18:1) [38] to SCD which then releases oleic acid to FABP4. The FA are then available to other enzymes involved in TAG synthesis.

### Membrane-associated ATP transporters

#### ABCG2 and its potential role in milk synthesis

We observed a 30-fold increase in *ABCG2 *[ATP-binding cassette, sub-family G (WHITE), member 2] transcript during lactation (Figure 2). mRNA abundance of this gene accounted for ~9% of total genes measured (Table 1). *ABCG2 *is a member of the large ATP binding cassette family of membrane-spanning efflux pumps that actively extrude a wide range of xenobiotics [42]. It is present in apical membrane of murine mammary alveolar epithelia and plays a role in active secretion of toxins into milk [42]. It also is present in the milk fat globule membrane (MFGM) [29], probably in the external bilayer originating from plasma membrane, due to its apical membrane localization. The large *ABCG2 *mRNA abundance and up-regulation, both in lactating bovine and murine mammary tissue [17,23], is biologically puzzling because the primary role of this transporter in other tissues is detoxification. Recently, it was reported that one amino acid substitution at position 581 (Tyr to Ser; Y581S) of *ABCG2 *resulted in decreased milk production but increased milk fat and protein concentration and yield [43]. Those data clearly do not support a role of *ABCG2 *in synthesis or secretion of milk fat. What seems apparent based on current bovine data is that *ABCG2 *plays an essential role in secretion of "some" important milk constituent [42]. Cholesterol transport was suggested [43] but it is not supported by the low amount [3] and pattern of cholesterol in bovine milk throughout lactation [44]. The only demonstrated role of *ABCG2 *in secretion of a milk component is for riboflavin, an essential, but quantitatively marginal, nutrient for the neonate [17]. Therefore, its large up-regulation is suggestive of other functions in milk synthesis besides riboflavin secretion.

The amount and pattern of cholesterol secretion into bovine milk agrees with the pattern of *ABCA1 *(ATP-binding cassette, sub-family A, member 1) transcript we observed (Figure 2). *ABCA1 *mRNA accounted for <1% of all genes measured (Table 1). This gene also has low expression in a number of other bovine tissues, and bovine mammary in particular [45]. *ABCA1 *is crucial for efflux of cholesterol from cells [46]. Taken together, data suggest a minor role of *ABCA1 *in bovine mammary cholesterol flux.

### Concerted action between *de novo* FA synthesis and desaturation in mammary TAG synthesis

#### ACACA, FASN, and *de novo* FA synthesis

**Figure 3:**
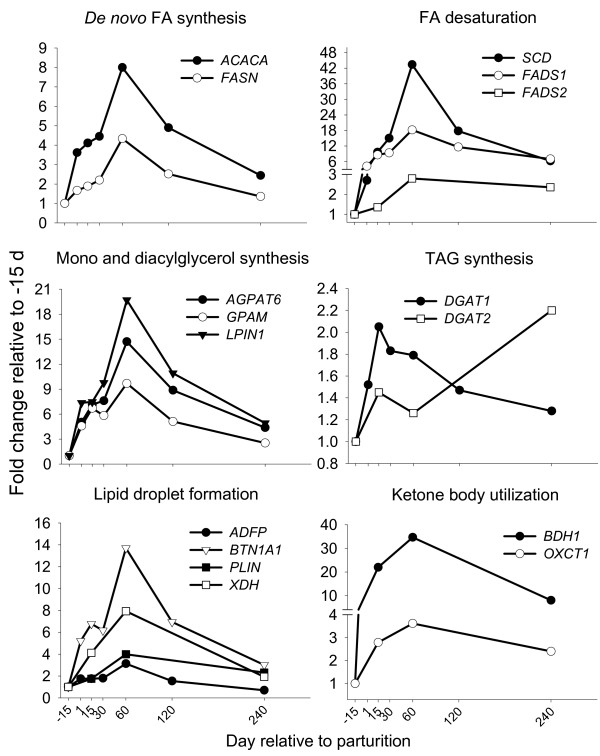
**Genes involved in *de novo *FA synthesis, LCFA desaturation, TAG synthesis, lipid droplet formation, and BHBA utilization**. Temporal expression patterns in bovine mammary of genes involved in *de novo *FA synthesis (*ACACA*, SEM = 0.62; *FASN*, SEM = 0.33), long-chain FA desaturation (*SCD*, SEM = 6.20; *FADS1*, SEM = 1.74; *FADS2*, SEM = 0.36), TAG synthesis (*GPAM*, SEM = 0.96; *AGPAT6*, SEM = 1.24; *LPIN1*, SEM = 3.9; *DGAT1*, SEM = 0.16; *DGAT2*, SEM = 0.86), lipid droplet formation (*ADFP*, SE = 0.23; *BTN1A1*, SEM = 1.10; *PLIN*, SEM = 1.29;*XDH*, SEM = 0.90), and ketone body utilization (*BDH1*, SEM = 8.60; *OXCT1*, SEM = 0.54). Statistical effect of time: *P *< 0.05 for all genes except *PLIN *(*P *= 0.17) and *DGAT2 *(*P *= 0.54).

Production of SCFA and palmitate from acetate is under control of ACACA, considered the rate-limiting step in *de novo *FA synthesis [2]. In subsequent steps, both, acetyl-CoA and butyryl-CoA (mostly from plasma β-hydroxybutyrate) are primers for the cytosolic multifunctional protein fatty acid synthase (FASN) [16]. The major product of FASN is palmitate but in ruminants the enzyme also produces SCFA [16]. In the present study, *ACACA *mRNA abundance accounted for <1% whereas *FASN *accounted for 7% of total genes measured (Table 1). However, *ACACA *mRNA had greater up-regulation during lactation compared with *FASN *(Figure 3). These data are consistent with activity values of the two enzymes from pregnancy through lactation in dairy cows [34]. Despite differences in magnitude, expression patterns among both genes were similar (r = 0.90; *P *< 0.01; see Excel File in Additional file 2), confirming previous findings summarized in a recent review article [6]. In fact, this was the case for several genes involved in TAG synthesis and transport. Clearly, bovine mammary lipid synthesis requires coordinate expression of several genes for TAG synthesis and secretion. This point was stressed in studies of rabbit mammary lipid metabolism several decades ago [47]. However, the scope of enzymes studied previously was relatively small compared with our study.

#### Stearoyl-CoA desaturase and milk TAG synthesis

Only a fraction of FA taken up by the mammary gland is unsaturated owing to extensive ruminal biohydrogenation. The primary enzyme involved in monounsaturated FA synthesis is stearoyl-CoA desaturase (SCD), which introduces a double bond in the Δ^9 ^position of myristoyl-, palmitoyl-, and stearoyl-CoA, primarily [48]. To date, two *SCD *isoforms have been identified and characterized in bovine: *SCD1 *and *SCD5*. *SCD1 *was first characterized in bovine adipose [49] and until the discovery of *SCD5*, it was believed to be the only *SCD *present in this species. Thus, the bovine gene is simply referred to as *SCD*. Bovine *SCD5 *has been identified and characterized only recently [50] and it is expressed almost exclusively in brain. The nucleotide sequence for a bovine *SCD6 *homolog A (*S. cerevisiae*) has been deposited at NCBI [51], but no characterization of this isoform has been performed to date. *SCD1 *has 33.5% and 31.3% global nucleotide alignment identity [52] with *SCD5 *and *SCD6*, respectively. The primer pair used in the present study (Additional file 1, Table S2) is specific only for *SCD1*. The *SCD *mRNA abundance was the highest (23%; Table 1) among all genes measured. The large *SCD *mRNA abundance, relative to other classical lipogenic genes (e.g., *ACACA*, *FASN*), and the >40-fold up-regulation during lactation (Figure 3) agrees with the suggestion by Kinsella, based on lactating mammary SCD activity [53], that it plays a crucial role in TAG synthesis.

All indexes of "apparent" desaturase activity increased during lactation except for stearic acid and palmitic acid desaturation. The latter index had a peak at the beginning of lactation followed by a dramatic decrease (Figure 1, bottom panel). The pattern of *SCD *mRNA was not significantly correlated with any of the Δ^9 ^desaturase indexes (Additional file 2), and was nearly opposite to the overall Δ^9 ^desaturase index (Table 2). A lack of correlation between desaturase indexes and SCD desaturase activity was found previously in bovine intramuscular fat [54], which along with our findings suggests that use of indexes is inappropriate for inferring *SCD *gene expression/activity at least when considering the lactation cycle [4]. Previous single-time point studies observed a positive correlation between *SCD *mRNA in goat mammary tissue and milk fat oleic acid [55] or desaturase indexes [56]. In our study, relative % mRNA abundance of *SCD *(23%; Table 1) was related to the total amount of Δ^9 ^FA (14:1*c*9 + 16:1*c*9 + 18:1*c*9 + *cis*9, *trans*11-18:1), which accounted for 19% of total milk FA (molar proportion; Additional file 1, Table S6).

Overall, our results support a central function of SCD in milk fat synthesis as previously demonstrated by intravenous infusions of sterculic acid, an inhibitor of SCD activity [57]. This point is supported, to some extent, by the recent discovery that both *SCD *and *DGAT1 *polymorphisms affect saturation level of milk fat and largely explain genetic variance on the desaturase indexes. The effect, however, was greater for *SCD *and primarily on medium/long-chain FA (from 10- to 16-carbon FA). Polymorphism in *DGAT1 *explained less variability but had a greater effect on long-chain FA (18-carbon) [58].

The lack of correlation between temporal desaturase indexes (i.e., apparent SCD activity) and *SCD *mRNA expression is not surprising due to the many factors that likely play a role in determining milk FA output. An important factor to consider is the selective uptake of stearic acid from blood VLDL by the mammary gland [59]. Mammary tissue relies heavily on utilization of VLDL-TAG during lactation [27]. Another key aspect is the high concentration of oleic acid, both in plasma at mid-lactation [60] and the NEFA pool at early lactation [61]. Oleic acid is the predominant FA in ER membranes [62] and Δ^9 ^desaturase is essential for their functional maintenance [48]. *FABP3 *expression and activity also could play a role in preferential channelling of palmitic and stearic acid for desaturation. Bovine FABP3 has a high affinity for both stearic and palmitic acid [38,63].

#### Very long-chain fatty acid desaturases

Synthesis of very-long-chain FA is carried out by fatty acid desaturase 1 (FADS1) and 2 (FADS2), which add double bonds at the Δ^5 ^and Δ^6 ^position of PUFA. Arachidonic acid (20:4n-6), eicosapentaenoic acid (20:5n-3), and docosahexaenoic acid (22:6n-3) are synthesized via FADS1 and FADS2 [64,65].* FADS1 *and *FADS2 *mRNA abundance was <1% of total genes measured (Table 1). The greater relative mRNA abundance of *FADS1 *compared with *FADS2 *is similar to the one reported in rat mammary [65]. Relative mRNA abundance in bovine mammary tissue was in concordance with the amount of product of both enzymes, which accounted only for 0.12% and 0.03% of total milk FA (Additional file 1, Table S6). *FADS1 *mRNA expression increased 18-fold by d 60 postpartum, whereas *FADS2 *mRNA increased only 3-fold (Figure 3). Expression pattern of *FADS1 *mRNA did not agree with the observed Δ^5 ^desaturase index, whereas *FADS2 *mRNA had a similar pattern compared with its index of activity (Additional file 1, Figure S2). In practical terms, it appears that *FADS1 *mRNA abundance/activity might be more amenable to dietary manipulation in order to increase omega-3/omega-6 ratio in milk fat.

### Formation of TAG and milk lipid droplets

#### Role for AGPAT6 and LPIN1 in TAG synthesis

Discrete steps in the pathway of TAG synthesis [66] have been discerned in classical lipogenic tissues (e.g., liver, adipose; [67]) and, despite lack of functional studies of mammary lipin, the same steps likely apply to the mammary gland. Expression of *GPAM *(glycerol-3-phosphate acyltransferase, mitochondrial), *AGPAT6 *(1-acylglycerol-3-phosphate O-acyltransferase 6), *DGAT1 *(diacylglycerol acyltransferase 1), and *LPIN1 *(lipin 1) mRNA accounted for >2%, >1%, ~0.1%, and ~0.1%, respectively, of total transcripts measured (Table 1). *DGAT2 *mRNA expression was nearly undetectable. Despite these differences, we observed that *LPIN1 *mRNA was up-regulated during lactation by 20-fold (Figure 3). The more abundant *GPAM *and *AGPAT6 *mRNA increased by 10- and 15-fold by d 60 post-partum (Figure 3). *GPAM *expression agrees with the greater enzyme activity in mammary gland during lactation in non-ruminants (e.g., [47]), and confirms its crucial role in TAG synthesis [16]. *AGPAT6 *and *LPIN1 *are the major isoforms within each gene family in bovine mammary tissue [19]. When the former was knocked out in lactating mice, they failed to synthesize milk fat [68].

Recent characterization of *AGPAT6 *showed that this gene is in fact the ortholog of human *GPAT4 *(microsomal glycerol-3-phosphate acyltransferase 4) [69]. The product of *GPAT4 *did not have AGPAT activity but instead a clear glycerol-3-phosphate acyltransferase activity [69]. However, authors failed to demonstrate an increase in TAG synthesis after overexpression of *GPAT4*. Despite these results in the mouse, our data support an important role of *AGPAT6 *in bovine mammary and very likely in TAG synthesis, as previously discussed [19]. Our data seem to suggest that AGPAT6 has AGPAT activity. This is inferred by the lower mRNA abundance and temporal increase in the transcript of other *AGPAT *in bovine mammary tissue [19], as well as the large up-regulation of *GPAM *expression. It seems unlikely that bovine mammary tissue would require a larger number of enzymes with GPAT activity relative to other downstream FA acylating enzymes.

A potential function of LPIN1 in regulation of transcription of other genes involved in milk fat synthesis cannot be excluded. Recently, it was demonstrated that LPIN1 is essential for PPARα [70] activation but it also interacts with PPARγ [71]. In addition, LPIN1 is a target of insulin-stimulated phosphorylation through mTOR [72], which in turn seems to promote microsomal vs. cytosolic localization of the protein. It could be possible that in bovine mammary tissue insulin signalling through INSR (insulin receptor) and IRS1 (insulin receptor substrate-1) as well as mTOR (*FRAP1*), all of which had a significant increase in mRNA expression (1.5-4-fold) through lactation [8], induces LPIN1 phosphorylation and localization to ER for DAG synthesis and TAG formation.

Relative mRNA abundance of *DGAT1 *was 17-fold greater compared with *DGAT2 *(Table 1) and had modest up-regulation particularly in early lactation (Figure 3). The temporal pattern in expression of this gene was similar to butyrate yield (Additional file 1, Table S7). DGAT1 acylates the *sn*-3 position of DAG and most butyrate in milk TAG is found here [44]. This protein has high affinity for butyryl-CoA and even higher for palmitoyl-CoA [73]. However, DGAT1 might favour use of butyrate for the *sn*-3 position of DAG as indicated by the larger affinity of AGPAT for LCFA in mammary [74] along with the preferential incorporation of palmitic acid in the *sn*-1 and *sn*-2 position [3].

The pattern of *DGAT1 *expression was unexpected because it is considered a QTL [75] for milk production traits, and is essential for murine mammary gland development and milk synthesis [76]. Data from the present study (i.e., fold-change and mRNA abundance) suggest that DGAT1, compared with other genes involved in TAG synthesis, is of minor importance in the overall process of milk fat synthesis. The temporal decrease or lack of increase in expression of *Dgat1 *in mammary tissue of FVB mouse [23] provides additional support. We do not, however, believe our findings contradict previous functional studies [77], demonstrating a pivotal role for DGAT1 in increasing milk TAG. The fact remains that DGAT1 is one of many proteins composing the TAG synthesis pathway [67]. A lack in functionality of any gene in this pathway can likely reduce the efficiency of TAG synthesis. Protein expression and functional studies during the entire lactation should be conducted to clarify the importance of DGAT, and others, in mammary lipid synthesis.

#### Milk lipid droplet formation

Milk fat globules are formed in the ER membrane via incorporation of newly-formed TAG, transported to the apical membrane, and eventually released [3]. Well-defined proteins involved in these processes in mammary include butyrophilin (*BTN1A1*), xanthine dehydrogenase (*XDH*), and adipophilin (*ADFP*) [3,15,29]. Relative mRNA abundance among these genes confirms the large amount of the respective protein product found in MFGM [3]. *ADFP *mRNA abundance (~10%) relative to other genes measured was almost twice that of *BTN1A1 *(~5%) (Table 1). Expression of *BTN1A1*, *XDH*, and *ADFP *increased during lactation and averaged 14-, 8-, and 3-fold by d 60 postpartum, respectively (Figure 3). The larger increase in expression of *BTN1A1 *seems to support a more crucial role for this gene, as recently suggested [78], in milk fat secretion compared with *XDH *and *ADFP*. However, despite the greater increase in mRNA expression for *BTN1A1 *than *ADFP *at 60 d post-partum relative to pre-partum, the larger overall abundance of *ADFP *transcript resulted in similar mRNA abundance for both genes at 60 d (data not shown). The similar proportion in relative mRNA of *BTN1A1 *and *ADFP *is in accordance with their protein abundance in mammary tissue [3]. Our results highlight the limitations of reporting gene expression data exclusively as *n*-fold change. Relative mRNA abundance also needs to be considered. The precise mechanism of milk fat secretion is still debated (e.g. [15,78]) but our data confirmed a role for *BTN1A1*, *XDH*, and *ADFP*. The similar pattern of expression and large correlation observed (r ≥ 0.92, *P *< 0.01; Supplemental Excel file) are indicative of a concerted action among the 3 genes and, thus, provides support for the tripartite model of murine milk lipid secretion [15].

Perilipins are a family of proteins localized in the periphery of intracellular lipid droplets and are essential for droplet formation as well as lipolysis in adipose tissue [79]. *ADFP *and the perilipin gene (*PLIN*) are both part of the perilipin family. Relative *PLIN *mRNA abundance was ~0.01% of total genes measured (Table 1) and its expression was only numerically up-regulated (~3-fold by d 60) during lactation. Our data do not support a significant role for *PLIN *in mammary lipid droplet formation. Functional studies could clarify the involvement, if any, of PLIN in this process.

#### BDH1 and OXCT1 and utilization of ketone bodies by mammary

β-hydroxybutyrate (**BHBA**) is the major ketone body produced in bovine species under most circumstances. Bovine mammary gland takes up large amounts of BHBA from blood [80]. Previous studies, focused mostly on *de novo *synthesis and oxidation of FA, concluded that use of BHBA (as 4-carbon units) by mammary cells is primarily for *de novo *FA synthesis. A minor portion is used as energy source through the TCA cycle, leaving unaccounted the fate of a substantial portion of the BHBA taken up [80]. BDH1 (3-hydroxybutyrate dehydrogenase, type 1) and OXCT1 (3-oxoacid CoA transferase 1) catalyze the initial and committed steps of BHBA utilization in mitochondria [81]. mRNA abundance for *BDH1 *and *OXCT1 *accounted for <0.1% of genes measured (Table 1). The large increase in expression and relative mRNA abundance during lactation (Figure 3; Table 1) correspond with their enzymatic activity level in lactating rat mammary tissue [82]. Based on these data, and a previous microarray study [8], we propose that the major fate of BHBA in bovine mammary is the synthesis of citrate (Additional file 1, Figure S3 for model and details).

### Transcription factors and nuclear receptors during lactation: potential roles in mammary lipid metabolism

#### SREBF-related networks and TAG synthesis

A large body of evidence supports the suggestion that SREBP1 (sterol regulatory element-binding protein 1) is pivotal in the regulation of milk fat synthesis in mouse [7] and cow [83]. SREBP1 and 2 reside as inactive precursors in the ER membrane and are transported to the Golgi for proteolytic cleavage (i.e. activation) prior to entering the nucleus and activation of sterol responsive element-containing genes (e.g., *ACACA*, *FASN*). The transport step to the Golgi is blocked by sterols via the sterol-sensing protein SCAP (SREBP cleavage activating protein). SCAP is essential for the movement of SREBP isofoms from the ER to the Golgi, essentially acting as gate keeper for movement of inactive SREBP1 and 2 [84]. Insulin induced gene (INSIG) 1 and 2 are proteins that interact with SCAP in an oxysterol-dependent and independent fashion and regulate the responsiveness of SREBP1 and 2 processing via SCAP, thus altering rates of lipogenesis [84].

**Figure 4:**
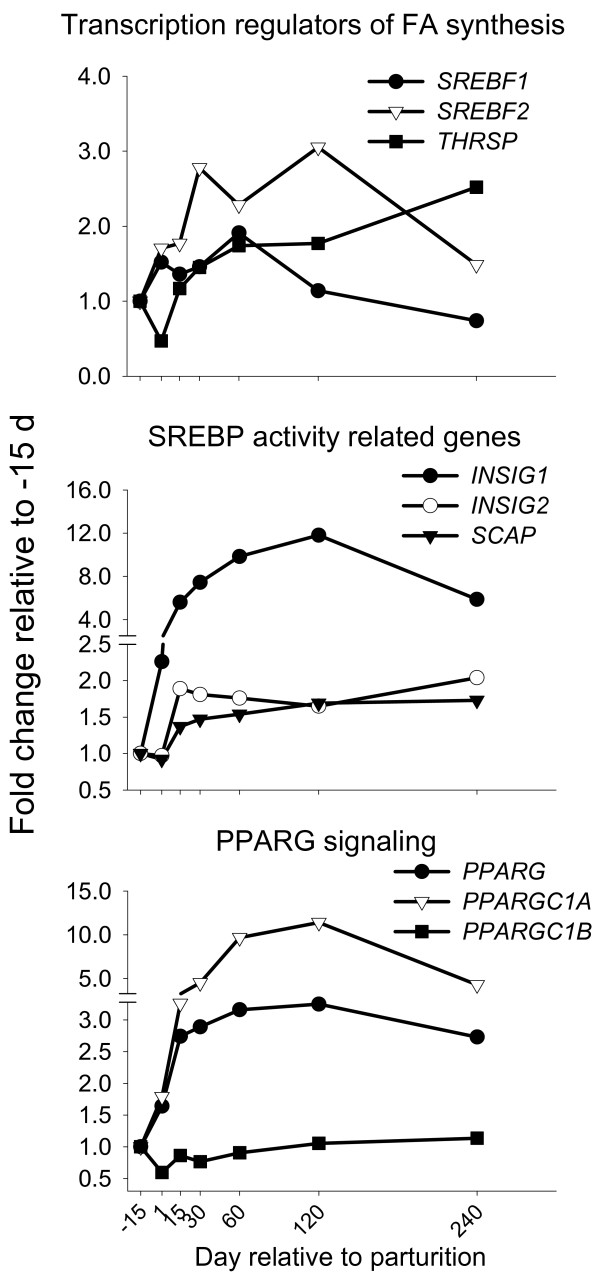
**Regulation of transcription in bovine mammary**. Temporal expression patterns in bovine mammary of genes involved in regulation of transcription (*SREBF1*, SEM = 0.18; *SREBF2*, SEM = 0.36; *THRSP*, SEM = 0.49; *INSIG1*, SEM = 1.30; *INSIG2*, SEM = 0.18; *SCAP*, SEM = 0.08; *PPARG*, SEM = 0.43; *PPARGC1A*, SEM = 6.6; *PPARGC1B*, SEM = 0.09). Statistical effect of time: *P *< 0.05 for all genes

Expression of SREBP genes (*SREBF1 *and *SREBF2*), and thyroid hormone responsive SPOT14 (*THRSP*) averaged ~2-fold by day 60 postpartum (Figure 4) but relative mRNA abundance was ~0.13% for *SREBF1 *and *SREBF2*, and only 0.01% of total genes measured for *THRSP *(Table 1). There are two isoforms of SREBP1 (a and c) that can be expressed at different levels in tissues. The two isoforms differ by only 84 nucleotides at the first exon [85] and each appears to be specific in the control of transcription of genes involved in cholesterol (isoform a) or TAG synthesis (isofom c) [86]. The primer pair used in our study is unable to differentiate between these because it amplifies mRNA at the 14^th ^exon.

*INSIG1, INSIG2*, and *SCAP *mRNA abundance accounted for ~0.4%, ~0.1%, and ~0.1%, respectively, of total genes studied (Table 1). These genes had increased mRNA expression during lactation, ranging from ~1.5-fold for *SCAP *to ~12-fold for *INSIG1 *at or close to peak lactation (Figure 4). It is apparent from our comprehensive analysis of *SREBF1*, in concert with its co-factors (*SCAP*, *INSIG1 *and *2*), that the complete activation program of SREBP is up-regulated in the bovine mammary gland during lactation. More *INSIG1 *mRNA was expressed in mammary tissue than *SCAP *or *SREBF1 *and *2 *(Table 1), and its level of up-regulation reached 10-12-fold between peak through mid-lactation (Figure 4). If this pattern of *INSIG1 *mRNA expression extended to the protein level, more SREBP might be retained in the ER via SCAP-INSIG1 binding (i.e., rendered inactive) [84].

INSIG1 binds oxysterols (not cholesterol) specifically and this specificity is directly correlated with the ability of these compounds to inhibit SREBP cleavage [84]. Data on oxysterol concentration/synthesis in bovine mammary tissue are not readily available but considering that mammary tissue uptake and synthesis of cholesterol is small we can infer low presence of these compounds. In this regard, it is interesting that genes coding for oxysterol binding proteins were up-regulated during lactation (discussed below). Cleavage inhibition of SREBP also can occur in the absence of sterols, particularly when the ratio of INSIG1/SCAP increases as in the present study [84]. Therefore, it appears that an increase of INSIG1 alone can be enough to block SREBP cleavage. In fact, decreased SREBP activity as a consequence of increased *INSIG1 *transcript has been observed in liver when *INSIG1 *is overexpressed [87] or during high fat diet-induced *INSIG1 *up-regulation [88]. Based on previous results and our data we postulate that mammary lipid synthesis cannot rely solely on transcriptional regulation via SREBP1 which is probably inhibited or controlled by INSIG1.

The observed up-regulation of *INSIG1 *during lactation apparently does not make teleological sense because mammary tissue requires substantial up-regulation of genes involved in *de novo *lipid synthesis (Figure 1; Table 2). It is striking, however, that *INSIG1 *mRNA was at its peak (d 120) when the cows likely were in positive energy balance, i.e. high blood insulin/glucagon, and the ratio of *de novo*-synthesized FA to imported FA was maximal (Figure 1). *INSIG1 *expression was positively correlated with the ratio of synthesized/imported FA (r = 0.38, *P *< 0.05; Additional file 2). Paradoxically, these data suggest some involvement of INSIG1 in inducing FA synthesis. This suggestion is supported by recent data [83], where a decrease of *INSIG1 *during experimentally-induced milk fat depression in lactating cows was observed.

Several factors could drive the marked increase in *INSIG1 *mRNA during lactation. For example, up-regulation of *INSIG1 *expression might be a consequence of *SREBF *isoform mRNA up-regulation and increased activity (i.e., induction of gene expression) of the corresponding proteins. SREBP1a and SREBP2 directly regulate *INSIG1 *gene expression [84]. Given the lipogenic capacity of mammary tissue, it is more likely that SREBP1c is the more abundant isoform. Thus, *INSIG1 *up-regulation in bovine mammary tissue could be under control of *SREBF2 *(Figure 4). Another reason for marked *INSIG1 *mRNA up-regulation might be its very short half-life [84], or as a necessary mechanism to sense low mammary cholesterol levels in order to regulate *de novo *FA synthesis.

Our data support a need of INSIG1 in controlling the induction of gene expression by *SREBF *isoforms. Therefore, *INSIG1 *could play a central role in orchestrating lipid metabolism (i.e., TAG vs. cholesterol) in bovine mammary tissue during lactation. In support of this, it previously has been suggested that high levels of INSIG1 create a situation in which low levels of endogenous sterols can trigger SCAP binding to INSIG1 without the necessity for exogenous sterols [84]. The "brake" effect of INSIG1 on TAG accumulation has been clearly demonstrated in mouse adipose tissue [88], when overexpression of *INSIG1 *in 3T3-L1 cells led to a decrease in mRNA abundance of lipogenic genes (e.g. *Srebp1c*, *Acaca*, *Chrebp*, *Pparg*). Our data, however, do not support a similar effect of INSIG1 on expression of mammary lipogenic genes in ruminant.

*SREBF2 *expression was up-regulated to a greater extent than *SREBF1 *expression during lactation (Figure 4). The reason for up-regulation of *SREBF2 *expression, and the similar mRNA abundance compared with *SREBF1*, is not apparent because this gene is thought to be involved primarily in cholesterol biosynthesis [85] and the amount of cholesterol in milk is low [89]. Cholesterol is almost exclusively synthesized *de novo *in bovine mammary tissue [90], thus *SREBF2 *expression also might be necessary to meet cholesterol requirements for MFGM formation [3]. In support of this we observed, via microarray analysis, that expression of several genes involved in cholesterol synthesis was significantly up-regulated (1.5-2-fold compare to -30 d) during lactation [8]. An additional function of SREBP2 is to induce mRNA expression of genes involved in FA synthesis [85]. It could be possible that *SREBF2 *mRNA up-regulation during lactation might compensate for the potential inhibition of INSIG1 on both SREBP.

#### Nuclear receptors and the lipogenic program

Genes involved in FA transport such as *LPL*, *CD36*, and *ACSL1 *are peroxisome proliferator-activated receptor gamma (PPARγ) target genes [91]. In the lactating mouse, expression of *LPL *and *ACSL1 *was up-regulated significantly despite the fact that expression of PPARγ was down-regulated [23]. However, it has recently been demonstrated that changes in abundance of adipocytes at several stages of pregnancy in murine mammary tissue (i.e., high in early pregnancy vs. low in late pregnancy) could account for the decrease in PPARγ gene (*PPARG*) mRNA abundance [92]. In bovine mammary, *PPARG *mRNA accounted only for 0.01% of total genes measured (Table 1) but was consistently up-regulated during lactation (Figure 4). The low mRNA abundance of "adipocyte-specific" genes (e.g., *DGAT2*, *PPARG*, *ACBP*, and *PLIN*), particularly at the end of pregnancy (i.e. -15 d), clearly indicates that biopsied cow mammary tissue contained low amounts of adipocytes. Thus, our longitudinal data on *PPARG *expression should represent that of epithelial cells. With this premise and despite the low mRNA abundance, up-regulation of *PPARG *mRNA during lactation suggests a potential role of this nuclear receptor in milk fat synthesis. A recent study with PPARγ-knockout mice indicated that absence of *PPARG *increased utilization of FA for synthesis of inflammatory lipids due to reduced TAG synthesis [93]. A role of *PPARG *in regulating the entire bovine milk fat synthesis machinery also is supported by recent results from our laboratory where treatment of MacT cells (immortalized bovine mammary epithelial cells) with rosiglitazone, a specific PPARγ agonist, resulted in coordinated up-regulation in expression of genes involved in FA import (e.g., *CD36*), *de novo *FA synthesis (e.g., *ACACA*, *FASN*, *SREBF1*), and TAG synthesis (e.g., *LPIN1*, *SCD*) [94].

The relative mRNA abundance for the PPAR gamma coactivators, *PPARGC1A *and *PPARGC1B*, was 0.04% and 0.01% of total genes measured (Table 1). Whereas expression of *PPARGC1A *was substantially up-regulated through d 120 post-partum (~11-fold), expression of *PPARGC1B *was consistently down-regulated during the entire lactation (Figure 4). Differences in relative mRNA abundance, large temporal up-regulation of *PPARGC1A *mRNA, and down-regulation of *PPARGC1B *transcript, suggest an important role of the former in bovine milk fat synthesis. The importance of *PPARGC1A *in the overall process of mammary lipid synthesis likely is more related to its well-defined role in regulating mitochondrial biogenesis and energy metabolism [95]. Up-regulation of *PPARGC1A *during lactation agrees with reported increases in numbers and turn-over rate of mitochondria in lactating mammary tissue [21]. Furthermore, our combined data (Table 1, Figure 4) also point to a concerted action of *PPARGC1A *and *INSIG1*. This last observation is captivating based on previous observations in murine [96] and bovine mammary epithelial cells [94] where *INSIG1 *was demonstrated to be a PPARγ responsive gene, suggesting that PPARG in mammary tissue could serve as regulator of SREBP activity.

### Ceramide-synthesis genes in bovine mammary tissue

#### Synthesis of Ceramide in bovine mammary

Ceramide, which is involved in cell signaling, cell cycle, and regulation of protein transport from ER to Golgi, is one of the most studied sphingolipids in nature [97,98]. Other sphingolipids with signaling roles include sphingosine (**Sph**) and sphingosine-1-phosphate (**S1P**) [98]. Sphingomyelin synthesis from ceramide is important for milk quality because this compound is considered a functional food component [16]. Although minor compared with TAG, sphingolipids are the third most important lipid component [99] in bovine milk fat. MFGM formation relies on sphingolipid and cholesterol availability [100], thus coordinated synthesis of both compounds is pivotal to milk lipid droplet formation/secretion. Sphingolipids are involved in lipid synthesis regulation through their action on SREBP [101]. Mammary tissue synthesizes sphingolipids *de novo *[99] from palmitoyl-CoA, leading to ceramide formation and incorporation into sphingomyelin [99]. Thus, palmitic acid used for ceramide synthesis in mammary appears a required step and also might represent a regulatory point for FA synthesis because ceramides can inhibit this process by blocking the activity of AKT/PKB [102]. Regulation of FA synthesis by sphingolipids in mammary tissue has never been investigated. Thus, we selected genes crucial in ceramide synthesis/degradation, as well as enzymes involved in Sph and S1P synthesis (Additional file 1, Figure S5) to explore further their role in lipid synthesis regulation.

**Figure 5:**
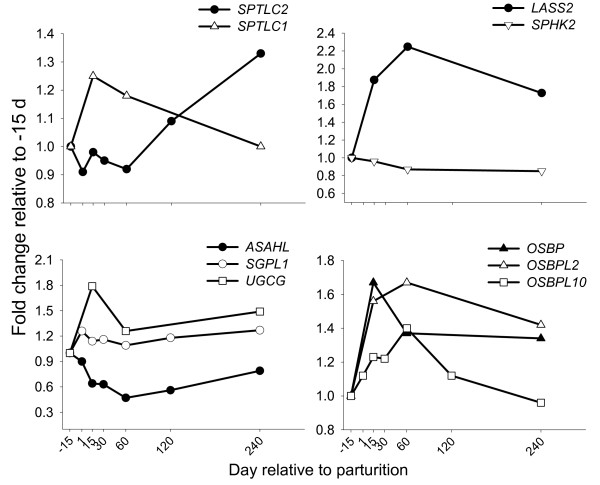
**Genes involved in sphingolipid synthesis in bovine mammary**. Temporal expression patterns in bovine mammary of genes involved in sphingolipid synthesis (*SPTLC1*, SEM = 0.10; *SPTLC2*, SEM = 0.08; *LASS2*, SEM = 0.14; *SPHK2*, SEM = 0.10; *ASAHL*, SEM = 0.10; *SGPL1*, SEM = 0.13; *UGCG*, SEM = 0.17; *OSBP*, SEM = 0.13; *OSBPL2*, SEM = 0.10; *OSBPL10*, SEM = 0.09). Statistical effect of time: *P *< 0.05 for all genes except *SGPL1 *(*P *= 0.63) and *SPHK2 *(*P *= 0.65).

In accordance with the minor concentration of sphingolipids in milk, genes in this pathway had low mRNA abundance ranging from 0.05% (N-acylsphingosine amidohydrolase-like or *ASAHL*) to 0.61% (LAG1 homolog, ceramide synthase 2 or *LASS2*) of total genes examined (Table 1). *LASS2 *was the most abundant among sphingolipid-related genes and the only one with >2-fold up-regulation during lactation (Figure 5). *LASS *isoforms are orthologues of the yeast Longevity-assurance gene. The enzyme is localized in the ER [97] and isoforms appear to have specific tissue distribution, suggesting they perform "specialized" functions [19,97]. *LASS2 *had peak expression at 60 d postpartum, in agreement with previous data on milk sphingolipid concentration [99]. Combined data on genes associated with ceramide synthesis suggest an increase in synthesis coupled with decreased degradation throughout lactation (Figure 5 and Additional file 1, Figure S5). An increase in mammary ceramide synthesis might potentially serve as a positive signal for proteins involved in lipid synthesis through activation of SREBP1 as suggested previously [101]. Increased sphingolipid synthesis during lactation also could affect availability of cholesterol for MFGM, and might explain the inverse pattern between milk cholesterol ester and sphingolipid [99].

#### Sterol and ceramides and their role in mammary lipid synthesis

Transport of ceramide from ER to Golgi is achieved by oxysterol binding proteins (OSBP), which also act as sterol sensors whose function is to integrate cellular sterol status with sphingomyelin metabolism [103]. A novel role for *OSBP *in regulation of lipid synthesis was demonstrated when overexpression of *OSBP *led to increases in TAG synthesis in mouse liver and concomitant up-regulation of *SREBF1 *and *INSIG1 *[104]. mRNA abundance of *OSBP *and related genes (*OSBPL2*, *OSBPL10*) in mammary was comparable with that of *SREBF1 *and *2*, and their expression was up-regulated ~1.5-fold during lactation (Figure 5) suggesting a functional role, likely involving the regulation of *SREBF1 *and the coordination of sphingolipid and cholesterol synthesis.

### Transcription regulation networks and proposed milk fat synthesis model

**Figure 6:**
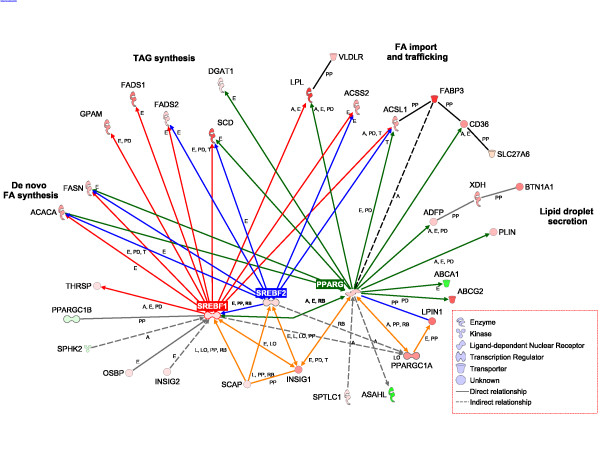
**Networks among genes involved in milk fat synthesis**. Networks were developed with Ingenuity Pathway Analysis^® ^(Ingenuity Systems, ) and edited to incorporate results from the present and previous studies in bovine mammary tissue. Red nodes denote positive fold changes and green nodes negative fold changes in expression at 60 relative to -15 d. Red, blue, and green edges denote genes whose transcription is under the control of *SREBF1*, *SREBF2*, and *PPARG*, respectively. Highlighted in orange is the network encompassing *PPARG*, *PPARGC1A*, *LPIN1*, *INSIG1*, and *SCAP *which controls expression/function of *SREBF *proteins. Letters along the edges denote effects on activity (A), expression (E), localization (LO), proteolysis (L), RNA binding (RB), protein-DNA binding (PD), and protein-protein binding (PP). Genes are grouped based on their primary function during milk fat synthesis.

We used our data set to develop gene networks using IPA (Figure 6). Details of networks are available in Additional file 1 (Supplementary Materials and Discussion) and the legend of Figure 6. The resulting networks clearly underscore a central role for *SREBF1*, *SREBF2*, and *PPARG *in controlling transcription of most of the genes assessed in the present study which coordinately regulate milk fat synthesis. The network also highlights a putative role of *PPARG*, in coordination with *PPARGC1A *and *INSIG1*, in controlling function/expression of *SREBF1 *(highlighted with orange edges). Thus, our data challenge the notion that *SREBF1 *is the central player regulating lipid synthesis in bovine mammary tissue. We propose that a network of transcription regulators and nuclear receptors, including *SREBF1*, *SREBF2*, *PPARG*, *INSIG1*, and *PPARGC1A*, coordinate activation of the genes driving the lipid synthesizing machinery (Figure 6). More functional studies are clearly needed to determine whether long-term up-regulation of transcription factors and nuclear receptors are determinant in inducing and maintaining milk fat synthesis. The specific roles of *INSIG1 *and *PPARG *during lactation need to be determined. Besides transcriptional regulation, other regulatory steps can determine the activity of the protein. For example, short term regulation of ACACA activity also occurs at the post-translational level [105].

**Figure 7:**
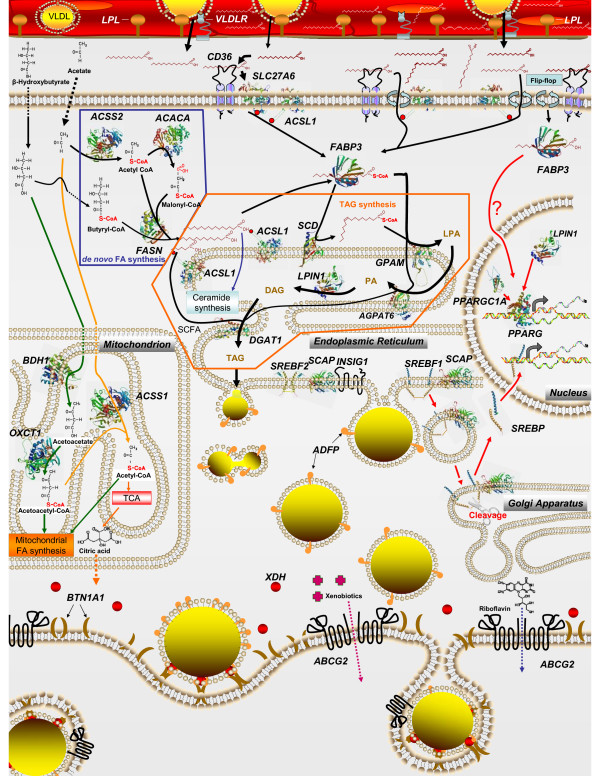
**Interrelationships among cellular pathways regulating milk fat synthesis in bovine mammary tissue**. Detailed description of the model is reported in the discussion section. Protein structures, when available, are the most updated from ModBase .

Taken together, our findings allowed for the development of an up-to-date model of milk fat synthesis regulation in bovine mammary tissue (Figure 7). The model incorporates the most recent information available, including our data, on enzymes involved in milk fat synthesis (i.e., subcellular location, fate of FA, and putative role). Following is the description of the model presented in Figure 7:

◦ **Endothelial long-chain FA (LCFA) transport: **the VLDL TAG core (or chylomicrons) is hydrolyzed by LPL in cooperation with VLDLR, which anchor the lipoprotein molecule and releases LCFA for transport across the endothelium into the extracellular space. LCFA are imported into mammary cells via flip-flop (passive or driven by "flippases") and protein-mediated mechanisms. CD36 appears to be the most important protein regulating LCFA import, and it might work in concert with FATP6 (SLC27A6). Once LCFA cross the membrane, most are activated into an LC-acyl-CoA (**LCACoA**) primarily via ACSL1 (activation shown by small red dots). Formation of LCACoA essentially traps LCFA inside the cell and activates them for subsequent utilization. LCACoA are captured primarily by FABP3, which transports them to specific intracellular organelles for utilization.

◦ **FA channelling and metabolic fates: **LCFA or LCACoA bound to FABP3 likely have three primary fates: 1) serve as substrate for SCD, which inserts a double bound at Δ^9 ^position of the LCFA (primarily 16:0 and 18:0), and the endogenous FA (primarily oleic acid) is subsequently transported by FABP4 to enzymes involved in TAG synthesis; 2) serve directly as substrate for TAG synthesis via sequential reactions carried out by GPAM (LCACoA ⇒ *sn*-1 of Glycerol-3-P to form lysophosphatidic acid [**LPA**]), GPAM in the present model is located on the ER instead in the mitochondria, where the proper location for the product of this gene is. Previous studies have demonstrated larger microsomal compared with mitochondrial GPAT activity in lactating bovine mammary tissue [106]. To date, however, only the mammalian mitochondrial GPAT have been annotated and characterized. The insertion of GPAM in the ER attempts to account for this limitation and to simplify the model. Other enzymes important for TAG synthesis include AGPAT6 (LCACoA ⇒ *sn*-2 position of LPA to form phosphatidic acic [**PA**]), LPIN1 (cleaves the P group of PA to form diacylglycerol [**DAG**]) and to a less extent, DGAT1 (LCACoA ⇒ *sn*-3 position of DAG to form **TAG**). The TAG pathway is denoted by black arrows and encircled by an orange line; 3) can regulate transcription via PPARG (see below).

◦ **Lipid droplet formation: **once TAG is formed it is inserted into the intra-leaflet of the ER membrane to form lipid droplet. In bovine mammary, ADFP (orange shape) is central for the formation of lipid droplets and for the secretory pathway involving BTN1A1 (brown half moon). The XDH (red circle) also seems to play a role in the mechanism encompassing ADFP and BTN1A1.

◦ ***De novo *FA synthesis, activation, channelling, and formation of TAG and phospholipid: ***de novo *FA synthesis (encircled in blue) is carried out by ACACA and FASN utilizing acetyl-CoA and butyryl-CoA. Formation of acetyl-CoA from acetate is carried out by ACSS2. Once FA with >10-carbons are formed they are activated by ACSL1 and bound to FABP3, which allows the FA to enter into the TAG synthesis pathway. Short-chain FA (**SCFA**) are inserted into TAG via DGAT1. A portion of palmitate (both from *de novo *synthesis and import) can be utilized for sphingolipid synthesis through ceramide (Additional file 1, Figure S5).

◦ **Transcriptional regulation: **denoted by red arrows. We propose a role for FABP3 in activation of gene expression by FA through PPARγ. Besides up-regulation during lactation, a chief role of *PPARG *in mammary lipid metabolism is supported by the large up-regulation in expression of *PPARGC1A *and *LPIN1 *two important PPARγ co-factors. Regulation of genes involved in *de novo *synthesis might partly be under control of SREBP1 (*SREBF1*), which is bound to SCAP in the ER and it is transported to the Golgi where it is cleaved. Activated SREBP1 then enters the nucleus and could regulate gene expression. However, the observed up-regulation of *INSIG1 *(binds SCAP and blocks SREBP transport to Golgi) could dampen SREBP activity in bovine mammary during lactation or at the very least elicit tight regulation of SREBP activity. Our data, together with previous findings, highlight a possible role of *PPARG *in regulating SREBP activity through regulation of *INSIG1 *expression (Figure 6). Results support an overlapping role of SREBP2 in regulation of expression of gene involved in *de novo *FA synthesis. Alternate routes of acetate and butyrate utilization are denoted by green (BHBA) and orange (acetate) arrows. Up-regulation in expression of *ACSS1 *during lactation allow utilization of acetate in mitochondria for energy generation primarily; whereas, mRNA up-regulation of *BDH1 *and *OXCT1 *allow BHBA entry into mitochondria. Primary routes of BHBA use are citrate synthesis (Additional file 1, Figure S3) and mitochondrial FA synthesis (Additional file 1, Figure S4).

◦ **Membrane-associated transporters: **the marked mRNA abundance and up-regulation of *ABCG2 *during lactation suggest a pivotal role of this gene in milk synthesis/secretion. Demonstrated roles of ABCG2 protein in mammary tissue include secretion of riboflavin (blue dotted arrow) and xenobiotics (violet dotted arrow).

## Conclusion

Lactation was characterized by dramatic up-regulation in expression of genes associated with FA uptake from blood (e.g., *LPL*, *CD36*) and intracellular transport/channelling (e.g., *FABP3*). These adaptations were mirrored in milk FA profiles, showing that mammary uptake relative to *de novo *synthesis predominated in early lactation. Although of lower magnitude, lactation also induced up-regulation of mRNA of genes involved in activation of FA (e.g, *ACSL1*, *ACSS2*), *de novo *synthesis (e.g., *ACACA*, *FASN*), desaturation (e.g., *SCD*, *FADS1*), synthesis of TAG (e.g., *AGPAT6*, *GPAM*), lipid droplet formation (e.g., *BTN1A1*, *XDH*), and ketone body utilization (e.g., *BDH1*, *OXCT1*). Temporal expression of genes with well-defined roles in mammary lipid metabolism peaked at 60 d post-partum and to some extent followed the lactation curve.

We could deduce a central role in endogenous oleic acid synthesis via SCD for mammary TAG synthesis. However, there was no statistical correlation between expression patterns of genes involved in desaturation and (Δ^5^, Δ^6^, Δ^9^) desaturase indexes rendering their use to infer temporal enzyme expression/activity meaningless. Furthermore, expression data highlighted the importance of ketone body utilization, mitochondrial biogenesis and PPARγ activity (*PPARGC1A*), and lipid droplet formation (*BTN1A1*, *XDH*, *ADFP*) in the global scheme of milk fat synthesis and secretion (Figure 7). Novel findings included a likely role for *PPARG, LASS2*, *INSIG1*, *SREBF2*, and *OSBP *in regulating lipid synthesis and mammary intracellular equilibrium between cholesterol and sphingolipids.

The complexity of mammary molecular adaptations over time was underscored by gene network analysis (Figure 6) as well as the apparent interrelationships that must coordinate the overall process of milk fat synthesis and secretion (Figure 7). This point is further exemplified by the large number of annotated transcripts, among them several transcription factors, whose expression is markedly up-regulated during lactation [8] most of which have currently unknown functions in bovine mammary tissue.

## Abbreviations

ACE: acetyl-CoA incorporation during FA Elongation; BHBA: β-hydroxybutyrate; DAG: diacylglycerol; FA: fatty acid(s); LCFA: long-chain fatty acid(s); LPA: lysophosphatidic acid; MGFM: milk fat globule membrane; PA: phosphatidic acid; qPCR: real-time RT-PCR; SCFA: short-chain fatty acid(s); TAG: triacylglycerol(s)

## Authors' contributions

MB conducted qPCR analysis, data transformation, handling and statistical analysis of data, and drafted the manuscript. JJL collected mammary biopsies and milk samples, conceived and designed the study, participated in its coordination, and helped draft the manuscript. All authors read and approved the final manuscript.

## Supplementary Material

Additional file 1 **Supplementary Materials and Methods and Results and Discussion**. The file contain additional Materials and Methods (RNA extraction, PCR, and design and evaluation of primers and details about measurement of milk yield, milk composition and fatty acid analysis) accompanied by 4 tables which include cows features (**Table S1**), qPCR primers information (**Table S2) **and validation (**Tables S3 **and **S4**); the file contain also additional Results and Discussion about milk FA composition, accompanied by 3 tables (**Table S5 **for PCR features, **Table S6 **for mole/day and** Table S7 **for g/100 g of milk fatty acids with calculated indexes) and 6 figures which include: the entire curve of lactation and milk fat yield and % (**Figure S1**), Δ^5 ^and Δ^6 ^calculated indexes (**Figure S2**), possible model of utilization of BHBA in bovine mammary (**Figure S3**); possible utilization of BHBA for mitochondrial FA synthesis (**Figure S4**), and possible model of phospholipid metabolism in bovine mammary during lactation (**Figure S5**). For each figure a detailed discussion is also provided in the caption. Click here for file

Additional file 2 **Excel file with correlation**. The file contains Pearson correlations among all measurements (genes and FA yield – mole/day). Correlations were analyzed using PROC CORR of SAS (SAS Inst. Inc. Cary, NC, release 8.0). The file contains 3 sheets with correlations and a legend. Click here for file
