# Supplementary material for: Gene networks driving bovine milk fat synthesis during the lactation cycle
Source: BMC Genomics. 2008 Jul 31;9:366. doi: 10.1186/1471-2164-9-366 (PMC2547860; doi:10.1186/1471-2164-9-366)
Supplement: Additional file 1 — Supplementary Materials and Methods and Results and Discussion. The file contain additional Materials and Methods (RNA extraction, PCR, and design and evaluation of primers and details about measurement of milk yield, milk composition and fatty acid analysis) accompanied by 4 tables which include cows features (Table S1), qPCR primers information (Table S2) and validation (Tables S3 and S4); the file contain also additional Results and Discussion about milk FA composition, accompanied by 3 tables (Table S5 for PCR features, Table S6 for mole/day and Table S7 for g/100 g of milk fatty acids with calculated indexes) and 6 figures which include: the entire curve of lactation and milk fat yield and % (Figure S1), Δ5 and Δ6 calculated indexes (Figure S2), possible model of utilization of BHBA in bovine mammary (Figure S3); possible utilization of BHBA for mitochondrial FA synthesis (Figure S4), and possible model of phospholipid metabolism in bovine mammary during lactation (Figure S5). For each figure a detailed discussion is also provided in the caption. [file 1471-2164-9-366-S1.pdf]

SUPPLEMENTARY MATERIALS and METHODS

RNA extraction and realt-time RT-PCR. Biopsy tissue was weighted (~0.5-1.0 g) and immediately subjected to RNA extraction using ice-cold Trizol (Invitrogen Corp., CA) as described previously [1]*. Genomic DNA was removed from RNA with DNase using RNeasy Mini Kit columns (Qiagen, Germany). RNA concentration was measured using a NanoDrop ND-1000 spectrophotometer (www.nanodrop.com). The purity of RNA (A_{260}/A_{280}) for all samples was above 1.9. RNA integrity was assessed by electrophoretic analysis of 28S and 18S rRNA subunits. A portion of the RNA was diluted to 100 ng/µL using DNase-RNase free water prior to reverse transcriptase.

The cDNA was synthesized using 100 ng RNA, 1 µg dT18 (Operon Biotechnologies, AL), 1 µL 10 mM dNTP mix (Invitrogen Corp., CA), 1 µL Random Primers (Invitrogen Corp., CA), and 10 µL DNase/RNase free water. The mixture was incubated at 65°C for 5 min and kept on ice for 3 min. This step has the purpose to increment the binding between primers (included dT18) with RNA. A total of 6 µL of master mix composed of 4.5 µL 5X First-Strand Buffer, 1 µL 0.1 M DTT, 0.25 µL (50 U) of SuperScript™ III RT (Invitrogen Corp., CA), and 0.25 µL of RNase Inhibitor (10 U, Promega, WI) was added. The reaction was performed in an Eppendorf Mastercycler® Gradient using the following temperature program: 25°C for 5 min, 50°C for 60 min and 70°C for 15 min. The cDNA was then diluted 1:4 with DNase/RNase free water.

The qPCR was performed using 4 µL diluted cDNA combined with 6 µL of a mixture composed of 5 µL 1 × SYBR Green master mix (Applied Biosystems, CA), 0.4 µL each of 10 µM forward and reverse primers, and 0.2 µL DNase/RNase free water in a MicroAmp™ Optical 384-Well Reaction Plate (Applied Biosystems, CA). Each sample was run in triplicate and a 6 point relative standard curve plus the non-template control (NTC) were used (User Bulletin #2, Applied Biosystems, CA). The reactions were performed in an ABI Prism 7900 HT SDS instrument (Applied Biosystems, CA) using the following conditions: 2 min at 50°C, 10 min at 95°C, 40 cycles of 15 s at 95°C (denaturation) and 1 min at 60°C (annealing + extension). The presence of a single PCR product was verified by the dissociation protocol using incremental temperatures to 95°C for 15 s plus 65°C for 15 s.

The Ct data were analyzed and transform using the standard curve with the 7900 HT Sequence Detection Systems Software (version 2.2.1, Applied Biosystems, CA). The final data were normalized using the geometric mean of the three most stable genes among the ones tested as internal controls, as reported previously [2].

Design and evaluation of primers. Primer features are shown in Suppl.Table 2 and gene description in Table 1. Primers were designed using Primer Express (version 2.0 or 3.0) with min amplicon size of 80 bp (but when possible were preferred amplicons ≥ 100 bp) and limited 3' G+C (Applied Biosystems, CA). Major part of the primers sets were designed to fall across exon–exon junctions. Primers were aligned against publicly available databases using BLASTN at NCBI and UCSC's Cow (*Bos taurus*) Genome Browser Gateway (http://genome.ucsc.edu/cgi-bin/hgGateway). Prior to qPCR the primers were tested by a 20 µL PCR reaction using the same protocol described for qPCR except the final dissociation protocol. For the purpose we used a universal reference cDNA (RNA mixture from 5 different tissues) to be sure to identify the gene. Five µL of the PCR product was run in a 2% agarose gel stained with ethidium bromide. The

*List of references is reported at the end of Supplementary Materials

remained 15 µL were cleaned using QIAquick® PCR Purification Kit (QIAGEN) and sequenced at the Core DNA Sequencing Facility of the Roy J. Carver Biotechnology Center at the University of Illinois, Urbana-Champaign (Tables S3 and S4). Only primers that presented a single band at the expected size and the right amplification product (verified by sequencing) were used for real time RT-PCR. The accuracy of a primer pair was also evaluated by the presence of a unique peak during the dissociation step at the end of qPCR.

Table S1. Features, performance in previous lactation, and predicted transmitting abilities (PTA) of cows used in the study.

Cow	Lactation	Milk kg/y	Fat%	Fat kg/y	Protein %
7313	1	7,515	4.0	279	3.3
7257	1	10,098	3.1	311	2.9
7251	1	11,010	3.6	398	2.9
7136	2	8,832	3.5	310	3.4
7264	1	8,622	3.9	332	3.3
7273	2	10,524	4.1	426	3.0
Mean	1.33	9,434	3.7	343	3.1
SD	0.52	1,328	0.4	57	0.2

Table S1 (Cont.)

Cow	Protein kg/y	PTA milk	PTA$	PTA Fat	PAT prot
7313	259	-373	35	-31	0
7257	294	1081	66	5	26
7251	317	-42	-166	-29	-19
7136	300	-77	70	-23	8
7264	286	-220	42	-3	8
7273	320	-266	-70	-7	-9
Mean	296	17	-3.8	-14.7	2.3
SD	22	535	94.5	15.0	15.6

Table S2. GenBank accession number, hybridization position, sequence, amplicon size, and source of primers use to analyze gene expression by qPCR.

Accession #	Gene	Primers[1]	Primers (5'-3')[2]	bp[3]	Source
DQ059505	*ABCA1*	F. 972	CGGCGGCTTCTCTTGTATAGC	101	This manuscript
		R. 1072	TTCAAGCGTGAGCTGAAACG		
DQ825760	*ABCG2*	F. 50	GAGCCATAGGTTTCCACTGTGA	83	This manuscript
		R. 132	CCACAGCAGAAGAATCTCCATT		
AJ132890	*ACACA*	F. 3709	CATCTTGTCCGAAACGTCGAT	101	This manuscript
		R. 3809	CCCTTCGAACATACACCTCCA		
BC114181	*ACBP*	F. 55	AGGCTGATTTTGACAAGGCG	141	This manuscript
		R. 195	GATCTAACAGTGCTGGACACTCAATATC		
BC119914	*ACSL1*	F. 1929	GTGGGCTCCTTTGAAGAACTGT	120	[3]
		R. 2047	ATAGATGCCTTTGACCTGTTCAAAT		
AB046741	*ACSS1*	F. 1835	CCGATCAGGTCCTGGTAGTGA	90	This manuscript
		R. 1924	CTCGGCCCATGACAATCTTC		
BC134532	*ACSS2*	F. 1881	GGCGAATGCCTCTACTGCTT	100	This manuscript
		R. 1970	GGCCAATCTTTTCTCTAATCTGCTT		
BC102211	*ADFP*	F. 139	TGGTCTCCTCGGCTTACATCA	81	This manuscript
		R. 219	TCATGCCCTTCTCTGCCATC		
DY208485	*AGPAT6*	F. 171	AAGCAAGTTGCCCATCCTCA	101	[4]
		R. 271	AAACTGTGGCTCCAATTTCGA		
AW656293	*ASAHL*	F. 281	ATTTACCACGGCCGGAATCT	101	This manuscript
		R. 381	CCTGTGTAGGCAATCTGCCC		
CR455522	*BDH1*	F. 563	CCCACCACCAGTCTGAGCAT	101	This manuscript
		R. 663	CCCACTACTCTGCACCCCAA		
M35551	*BTN1A1*	F. 1099	AGGACGGACTGGGCAATTG	81	This manuscript
		R. 1179	GAACCCATTCTCGGGAGTCAT		
X91503	*CD36*	F. 743	GTACAGATGCAGCCTCATTTCC	81	This manuscript
		R. 823	TGGACCTGCAAATATCAGAGGA		
NM_174693	*DGAT1*	F. 177	CCACTGGGACCTGAGGTGTC	101	This manuscript
		R. 277	GCATCACCACACACCAATTCA		
BT030532.1	*DGAT2*	F.389	CATGTACACATTCTGCACCGATT	100	This manuscript
		R.488	TGACCTCCTGCCACCTTTCT		
DN518905	*FABP3*	F. 458	GAACTCGACTCCCAGCTTGAA	102	[4]
		R. 559	AAGCCTACCACAATCATCGAAG		
EE347846	*FADS1*	F. 552	GGTGGACTTGGCCTGGATG	101	This manuscript
		R. 652	TGACCATGAAGACAAGCCCC		
DV895683	*FADS2*	F. 94	AAAGGGTGCCTCTGCCAACT	101	This manuscript
		R. 194	ACACGTGCAGCATGTTCACA		
CR552737	*FASN*	F. 6383	ACCTCGTGAAGGCTGTGACTCA	92	This manuscript
		R. 6474	TGAGTCGAGGCCAAGGTCTGAA		
AY515690	*GPAM*	F. 1963	GCAGGTTTATCCAGTATGGCATT	63	[3]
		R. 2026	GGACTGATATCTTCCTGATCATCTTG		
XM_589325	*INSIG1*	F. 438	AAAGTTAGCAGTCGCGTCGTC	120	[5]
		R. 557	TTGTGTGGCTCTCCAAGGTGA		
XM_614207	*INSIG2*	F. 494	TCCAGTGTGATGCGGTGTGTA	109	[5]
		R. 602	TGGATAGTGCAGCCAGTGTGA		

1 Primer direction (F – forward; R – reverse) and hybridization position on the sequence

2 Exon-exon junctions are underlined

3 Amplicon size in base pair (bp)

*

Table S2 (Cont.)

Accession #	Gene	Primers[1]	Primers (5'-3')[2]	bp[3]	Source
BC103330	*LASS2*	F. 657	TGACGTCAAGCGAAAGGATTT	101	This manuscript
		R. 757	TCCCTGCTCGGACGTAATTG		
DV797268	*LPIN1*	F. 147	TGGCCACCAGAATAAAGCATG	101	[4]
		R. 247	GCTGACGCTGGACAACAGG		
BC118091	*LPL*	F. 327	ACACAGCTGAGGACACTTGCC	101	This manuscript
		R. 427	GCCATGGATCACCACAAAGG		
EH174150	*OSBP*	F. 342	GTGAGCAGGTGAGCCACCAT	111	This manuscript
		R. 452	GGTATTTGCCGCGAAACTTG		
BC102883	*OSBPL2*	F. 412	AGAAGTGCATCGGCTTGGAG	124	This manuscript
		R. 535	CGGGAGGCTCTGTGAATTAGG		
DT840936	*OSBPL10*	F. 320	GGAGGGAAAGTCAGCATCACC	101	This manuscript
		R. 420	GCTGTGACCCTGTGGACCTT		
CN441025	*OXCT1*	F. 186	CAATGCTAGGAGCCATGCAG	101	This manuscript
		R. 286	CACTAGATCCATAGCCCCTCCC		
DV814745	*PLIN*	F. 443	GATCGCCTCTGAGCTGAAGG	108	This manuscript
		R. 550	AGAGCGGCCCCTAGGATTT		
NM_181024	*PPARG*	F. 135	CCAAATATCGGTGGGAGTCG	101	This manuscript
		R. 235	ACAGCGAAGGGCTCACTCTC		
AB106107	*PPARGC1A*	F. 625	AAAAGCCACAAAGACGTCCG	101	[5]
		R. 725	TCTGCTGCTGTTCCGGTTCT		
CX951189	*PPARGC1B*	F. 213	GCCTCCTTCAGTAAGCTGTCAA	103	[5]
		R. 315	GGCCCCGCTATACTGACTATGA		
DV935188	*SCAP*	F. 990	CCATGTGCACTTCAAGGAGGA	108	[5]
		R. 1097	ATGTCGATCTTGCGTGTGGAG		
AY241933	*SCD*	F. 665	TCCTGTTGTTGTGCTTCATCC	101	This manuscript
		R. 765	GGCATAACGGAATAAGGTGGC		
EH163381	*SGPL1*	F. 657	GGCTTATGGAGATTTCGCATG	111	This manuscript
		R. 767	CCCCCATTGAATAGGGAACAA		
BC116169	*SPHK2*	F. 84	CCTCTCAGAGCCACAGACCAA	103	This manuscript
		R. 186	ACCATGTCAGCAAGACGCTG		
BC105250	*SPTLC1*	F. 458	GGCACATTTGATGTGCACTTG	101	This manuscript
		R. 558	CTGGCTATGGTGGCAAATCC		
DN518712	*SPTLC2*	F. 484	AGCAGTATCAAGCGTTTCTCTGGTAT	103	This manuscript
		R. 584	TTGGTGTAGTTGTGGTAGGATTTCC		
TC263657	*SREBF1*	F. 143	CCAGCTGACAGCTCCATTGA	67	[3]
		R. 209	TGCGCGCCACAAGGA		
DV921555	*SREBF2*	F. 4134	AGGTCTCTGGGCACCATGC	101	This manuscript
		R. 4234	CATCACCGCAACCCCAAG		
AY656814	*THRSP*	F. 631	CTACCTTCCTCTGAGCACCAGTTC	151	[5]
		R. 781	ACACACTGACCAGGTGACAGACA		
BC123602	*UGCG*	F. 846	TGACCGAGGTTGGAGGTTTG	101	This manuscript
		R. 946	TGGTCCACCTGATCATTCTGG		
AJ609502	*VLDLR*	F.68	GCCCAGAACAGTGCCATATGA	103	This manuscript
		R.200	TTTTCACCATCACACCGCC		
BC102076	*XDH*	F. 258	GATCATCCACTTTTCTGCCAATG	100	This manuscript
		R. 357	CCTCGTCTTGGTGCTTCCAA		

*

Table S3. Sequencing results using BLASTN from NCBI (http://www.ncbi.nlm.nih.gov/BLAST/Blast.cgi?PAGE=Nucleotides&PROGRAM=blastn&MEGABLAST=on&BLAST_PROGRAMS=megaBlast&PAGE_TYPE=BlastSearch&SHOW_DEFAULTS=on) against nucleotide collection (nr/nt).

Gene	Best hit in NCBI	Score
ABCA1	Bos taurus ATP-binding cassette sub-family A member 1	111.0
ABCG2	Bos taurus ATP-binding cassette, sub-family G (WHITE), member	99.0
ACACA	Bos taurus acetyl-coenzyme A carboxylase alpha (ACACA)	93.7
ACBP(DBI)	PREDICTED: Bos taurus similar to Acyl-CoA-binding protein (ACBP) (DBI) (Endozepine)	172.0
ACAS2L	Bos taurus acetyl-Coenzyme A synthetase 2 (AMP forming)-like (ACAS2L)	102.0
ACSS2	Bos taurus acetyl-CoA synthetase 2	134.0
ADFP	Bos taurus adipose differentiation-related protein	85.7
ASAHL1	Bos taurus hypothetical LOC515375 (85% SIMILAR TO Macaca ASAHL)	113.0
BDH1	Bos taurus 3-hydroxybutyrate dehydrogenase, type 1	111.0
BTN1A1	Butyrophilin, subfamily 1, member A1 [Bos taurus]	91.5
CD36	Bos taurus CD36 molecule (thrombospondin receptor)	86.0
DGAT1	Bos taurus diacylglycerol O-acyltransferase homolog 1 (mouse)	122.0
DGAT2	Bos taurus diacylglycerol O-acyltransferase homolog 2	95.1
FADS1	PREDICTED: Bos taurus similar to BC269730_2 (LOC533107) (FADS1)	111.0
FADS2	Bos taurus fatty acid desaturase 2	120.0
FASN	Bos taurus fatty acid synthase (FASN)	71.9
INSIG1	PREDICTED: Bos taurus similar to insulin induced gene 1, transcript variant 2	123.0
INSIG2	PREDICTED: Bos taurus similar to Insulin induced gene 2 (LOC534440)	129.0
LASS2	Bos taurus LAG1 homolog, ceramide synthase 2 (LASS2)	116.0
LPL	Bos taurus lipoprotein lipase, mRNA	123.0

*

Table S3 (Cont.)

Gene	Best hit in NCBI	Score
OSBP	PREDICTED: Bos taurus similar to Oxysterol-binding protein 1	105.0
OSBPL2	Bos taurus oxysterol binding protein-like 2, mRNA	140.0
OSBPL10	PREDICTED: Bos taurus similar to oxysterol-binding protein-like protein OSBPL10	116.0
OXCT1	Bos taurus similar to 3-oxoacid CoA transferase 1	102.0
PLIN	Bos taurus similar to adipocyte lipid droplet binding proteiN	134.0
PPARG	Bos taurus peroxisome proliferator-activated receptor gamma	107.0
PPARGC1A	Bos taurus peroxisome proliferator-activated receptor gamma, coactivator 1 alpha	123.0
PPARGC1B	PREDICTED: Bos taurus similar to PGC-1-related estrogen receptor alpha coactivator	170.0
SCAP	Predicted: Similar to sterol response element binding protein cleavage-activating protein	83.8
SCD	Bos taurus stearoyl-CoA desaturase (delta-9-desaturase)	79.8
SGPL1	Bos taurus similar to sphingosine-1-phosphate lyase	138.0
SPHK2	PREDICTED: Bos taurus hypothetical LOC533103 (LOC533103) [in UIUC as SPHK2)	96.9
SPTLC1	Bos taurus serine palmitoyltransferase, long chain base	118.0
SPTLC2	Bos taurus similar to serine palmitoyltransferase, subunit II	122.0
SREBF2	Predicted: Bos taurus similar to sterol regulatory element-binding transcription factor 2	136.0
THRSP	Bos taurus thyroid hormone-responsive protein	187.0
UGCG	Bos taurus similar to Ceramide glucosyltransferase (Glucosylceramide synthase)	100.0
VLDLR	Bos taurus very low density lipoprotein receptor	123.0
XDH	Bos taurus xanthine dehydrogenase, mRNA	107.0

GPAM and *SREBF1* were not sequenced due to the short PCR product (<70 bp), which requires cloning.

*

Table S4. Sequencing results obtained from PCR product.

Gene	Sequence
ABCA1	GCGACGCATGAAGGACATACGCAAAGTTTTGAAGACGTTATACCAGATCGAGGCGTTTCAGCTCACGCATTGAAA
ABCG2	CTAACCCTGCAGACTTCTTCCTGGACATCATTAATGGAGATTCTTCTGCTGTGGA
ACACA	TCTCGACTGGTTGCTGTGATAGAAGAAGTTTGGGTAGGACAGTCAAAAATCGACGTTTCGGACAAGATGGA
ACBP(DBI)	AAGACAGTCAGAGACTGAACAGGCCAGATGATGAGGAATTAAAAGAGCTCTATGGACTCTACAAACAGTCTGTAATTGGAGACATAGATATTGAGTGTCCAGCACTGTTAGATCA
ACAS2L	GCACTACTCGGTCTGGAAAAGTCATGCGGAGGCTCCTGAGGAAGATTGTCATGGGCCGAGA
ACSS2	CTGTGCGATGGCCACATCTTCAGCCCAGCTCTCACTGAGGAGCTCAAGAAGCAGATTAGAGAAAAGATTGGCCATCGCCCTG
ADFP	GATCTTCCTTACTTGAAGTCTCTGTGTGAGATGGCAGAGAAGGGCATG
ASAHL1	GGTAGATGTGAAGTTCTTACGCACTTGACTTTGGATGTACAGTTCATAAAGAATGGGCAGATTGCCTACACAGGATTT
BDH1	ACCAGCCCAATTTGCATACCCAGTGAATGGCTCAGGCCTTCTCAGTTGGGGTGCAGAGTAGTGGGAC
BTN1A1	GCAATTGCATCTGTAGGGAGAATAGTTGTGATGAAGAAAGGATTTGACCCCATGACTCCCGAGAATGGGTTCAACTTAAAGG
CD36	AGTTGAAGACAAGGGTATTGCAATTTTTCTCCTCTGATATTTGCAGGTCCA
DGAT1	CGCAGCGATCCCTGTTCAGTTCTGACAGTGGCTTCAGCAACTACCGTGGCATCCTGAATTGGTGTGTGGTGATGCACA
DGAT2	CTGTTACTTCACCTGGCTGGTGTTTGACTGGAACACACCCAAGAAAGGTGGCAGGA
FADS1	CTCCTCAGCTCGTATCCTTCCTCACTTATGTGCCGCTGTTGGGACTGAAAGGCTTCCTGGGGCTTGTCTTCAATGGTCAA
FADS2	GCATGCCTTCCAGCACCATGCCAAACCCAACATCTTCCACAAGGATCCAGATGTGAACATGCTGCACGTGTTAACCAT
FASN	GCGAGACGTCTAGGTGTACGGGTGCCAGTCACGGATGCCCAGGATGTGAGTCACAGCCTTCAACGAGGTAAG
INSIG1	CAGGTCAGCGCGAGTGGGCCAGCGTGATGCGCTGCGTGGCCGTCTTCGTTGGCATCAACCACGCTAGTGCATAAACTGGGAA
INSIG2	GAATTGAGATGGTCCAGTGTGATGCGGTGTGTAGCCGTCTTTGTTGGTATAAATCACGCCAGCGCTAAAGTGGATTTATACCAACAAAGACGGCTACACACCGCATCACACTGGACCATTCTCTTTT
LASS2	GCAAACTTCATCACGTGGCCACCATCATCCTCATCAGTTTCTCCTGGTTTGCCAATTACGTCCGAGCAGGGAA
LPL	GCACTGTGAAGGTCTCTGCCTGAGCTGCAGAAAGAACCGTTGCAACAACATGGGCTACGAGATCAACAAGGATT

*

Table S4 (Cont.)

Gene	Sequence
OSBP	CGTCCGTCGATCAGAACGGCTGGACGCTGCGGCAGGAGATCAAGATTACCAGCAAGTTTCGCGGCAAATACCA
OSBPL2	GCAACTAGTACATAGCCTTCAGTAGCCCCTGAGTTTCCTGCAGCGGATCACAGAGTACATGGAACATGTGTACCTAATTCACAGAGCCTCCCGA
OSBPL10	GATCAGCATCTCAGCCACCGTCATCTTCCACACGAAGCCTTTCTACGGGGGCAAGGTCCACAGGGTCACAGCAA
OXCT1	GCATGCTGACCTGGCGTACTGGATGATACCTGGAAAGATGGTGAAAGGAATGGGAGGGGCCTATGGATCCTAGTGC
PLIN	CTCACGCCTTCGCAGCGCCAGGAACAGCATCAGTGTGCCCATTGCCAGCACTTCAGACAAAATCCTAGGGGCCGCTCTACAGA
PPARG	TAACAGTACTCTCCTAAAATACGGCGTGCACGAGATCATTTACACGATGCTGGCCTCCTTGATGAA
PPARGC1A	GCTGCTTCTCAGTATCTGACCACAAATGATGACCCTCCTCACACCAAACCCACAGAGAACCGGAACAGCAGCAGATA
PPARGC1B	GCGAAGCGTGTTCAGACGGAACGCCAAGCGTCCGGCATGCCAGGAAGCGGCGGGAAAAGGCCATTGGCGAAGGCCGCGTGGTGTACGTTCGAAACCTGTCA
SCAP	GAGTATGGCGACAGGGAGTATGTAGGGTGGGTCACCAGGGGGGGGATGAGCTCAGCGATGCCAATCTCCTCCTTGAAGTGCACATGGGA
SCD	GACGGCAGGATCTGTGGATGAACGTTTCAAAACAGCCTGTTTTTTGCCACCTTATTCCGTATATAGCCAG
SGPL1	GCAGGCTAGTCATCCAGACATCTTCCCCGGACTCCGGAAGATAGAGGCAGAGATTGTGAGGATGGCTTGTTCCCTATTCAATGGGGAGA
SPHK2	CATAGCCGTACTCCTGCCATACCACCCACCCATGGCCTCCGAACCAGGCCCAGCGTCTTGCTGACATGGCTAA
SPTLC1	GGGTAACGCGTGGCAAAATTTATGAAGACAGAAGAAGCCATAATATACTCATATGGATTTGCCACCATAGCCAGGAA
SPTLC2	GGACGTCGACAGGCAAAATCTGGCTCTGGACTCAATGATTGGGGTAGCAGGAAATCCTACCACAACTACACCAAATG
SREBF2	ACAGACTCGAGGGCCAGTGGCCACTTGTGGAGCAGCCTCAACGTTAGTGGGGCCACCTGTGACCCCAACCTTAACCATGCCTGGTCCAGCAA
THRSP	CGTCATCTGCTCCGTTAGGCTGCCTGCTGCTGTTCACAACTCCTCACTCCTCTTACTAGCTTGGGGTCGGAAGCCAGTGATTCATGAGGGACCACATGTCTGTCACCTGGTCAGTGTGTA
UGCG	GCTACGACATCACGGTCGTCATGCAACTCTGGCTCATATTCAATTTCTCAATTTCAATCCAGAATGATCAGGTGGACCATACTTAGG
VLDLR	GCACTAAATGAAATCAGCTGTGGTGCCCGCTCCACTCAGTGTATCCCAGTGTCCTGGCGGTGTGATGGTGAACACAAAAG
XDH	CCGTCCTGGCTCCATCTGCACCTTGCACCACGTGGCTGTGACGACTGTGGAAGGAATTGGAAGCACCTACAGACTGCAGCGATT

*

Milk Yield, Milk Composition and Fatty Acid Analysis Milk yield was electronically recorded twice a day (each milking) during the entire lactating period, i.e. over 240 individual measurements for each cow (Figure S1, top panel). To take into consideration the daily variation in milk yield as well as the effect of tissue biopsy on milk production, data from the last 5 days before biopsy were averaged with data from 4 days post-biopsy disregarding production data from both the day of biopsy and the first day post-biopsy (Figure S1, bottom panel). This was deemed necessary because the biopsy procedure typically leads to a reduction in milk yield for the first 2-3 milkings [6]. Milk fatty acids and milk composition were analyzed as described previously [7].

*

SUPPLEMENTARY RESULTS and DISCUSSION

Figure S1. Top panel, milk yield during the entire lactation (>240 measurements) for the 6 cows under investigation. Bottom panel, milk yield and composition around biopsy times (see Suppl. Materials and Methods for details).

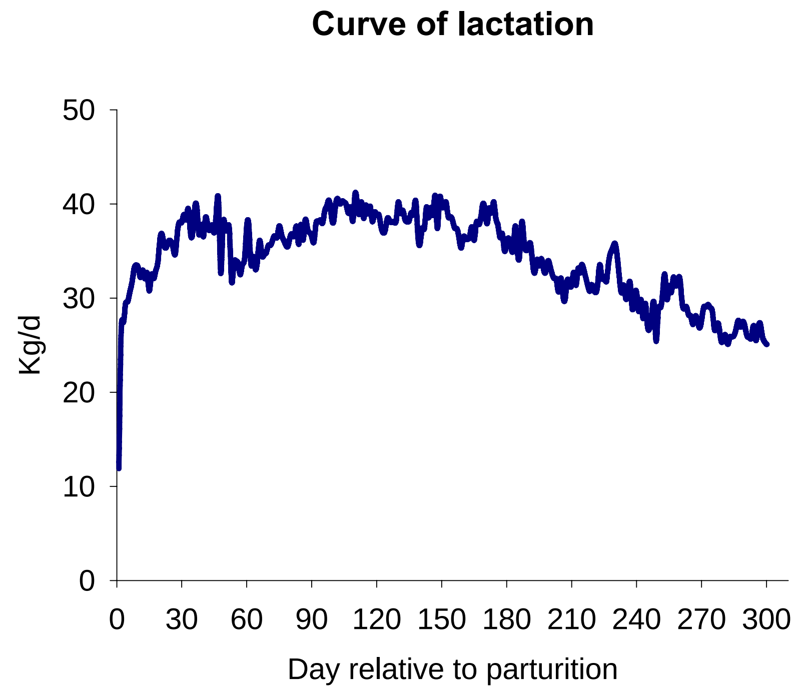

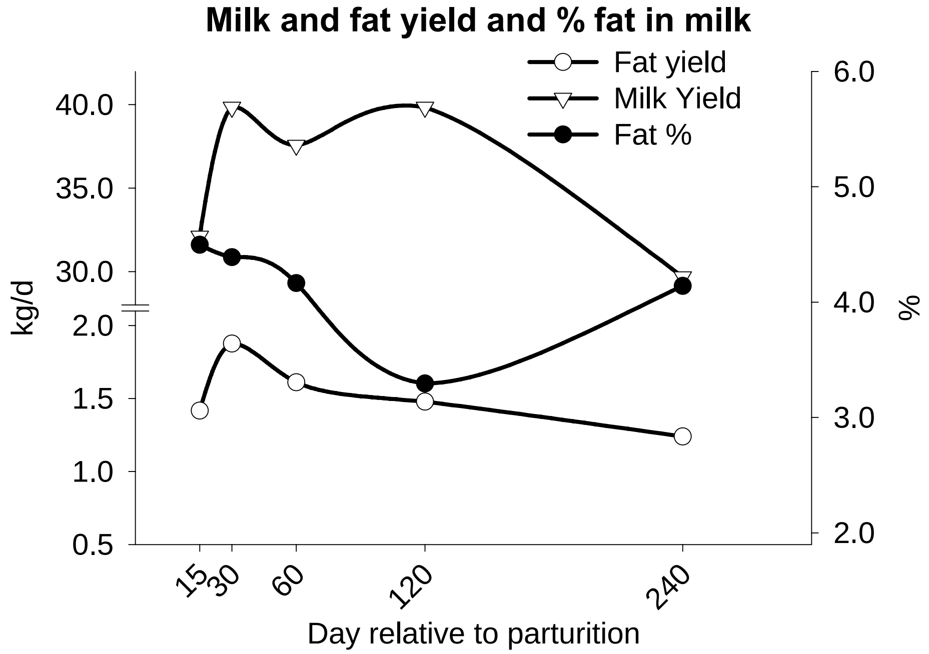

*

Table S5. qPCR performance data among the 45 genes measured.

Gene	Median Ct[1]	Median ΔCt[2]	Slope	R2[3]	Efficiency[4]
ABCA1	23.8	2.91	-2.99	0.995	2.16
ABCG2	19.9	-3.16	-2.89	0.998	2.22
ACACA	22.6	-0.35	-2.99	0.996	2.16
ACBP(DBI)	23.0	1.86	-3.00	0.996	2.15
ACSL1	20.7	-0.33	-2.96	0.994	2.18
ACAS2L	22.1	0.97	-3.01	0.992	2.15
ACSS2	21.0	0.21	-3.14	0.997	2.08
ADFP	19.5	-3.41	-2.99	0.994	2.16
AGPAT6	22.1	-0.79	-2.97	0.998	2.17
ASAHL	24.3	3.26	-2.95	0.994	2.18
BDH1	26.1	4.78	-3.01	0.993	2.15
BTN1A1	19.7	-2.43	-2.89	0.996	2.22
CD36	20.4	-2.45	-2.96	0.997	2.18
DGAT1	25.1	2.16	-3.08	0.998	2.11
DGAT2	29.3	8.40	-3.08	0.976	2.11
FABP3	19.0	-4.00	-2.96	0.996	2.18
FADS1	22.6	1.57	-2.95	0.993	2.18
FADS2	28.8	7.87	-3.18	0.992	2.06
FASN	19.8	-3.03	-3.00	0.999	2.15
GPAM	20.6	-1.61	-3.13	0.999	2.09
INSIG1	22.2	0.95	-3.25	0.998	2.03
INSIG2	24.2	3.06	-3.38	0.997	1.97
LASS2	21.2	0.18	-3.13	0.998	2.08
LPIN1	23.2	2.20	-3.01	0.995	2.15
LPL	19.6	-3.37	-2.95	0.998	2.18

[1] The median is calculated considering all time points and all animals
[2] The median of ΔCt is calculated from [Ct gene – Ct internal control] for each time point and each animal
[3] The R2 stand for the coefficient of determination of the standard curve
[4] The efficiency is determined by $[10^{(-1/\text{Slope})}]$

*

Table S5 (Cont.)

Gene	Median Ct[1]	Median ΔCt[2]	Slope	R2[3]	Efficiency[4]
OSBP	22.8	1.81	-3.18	0.999	2.06
OSBPL2	24.1	3.21	-3.05	0.993	2.13
OSBPL10	22.8	1.78	-2.94	0.996	2.19
OXCT1	24.0	3.07	-3.16	0.995	2.07
PPARG	28.5	5.50	-2.79	0.998	2.28
PPARGC1A	26.4	5.48	-2.86	0.997	2.24
PPARGC1B	26.7	3.64	-3.37	0.993	1.98
PLIN	26.5	5.91	-3.05	0.992	2.13
SCAP	23.6	2.26	-3.10	0.996	2.10
SCD	17.3	-4.74	-2.86	0.997	2.24
SGPL1	24.2	3.19	-3.04	0.990	2.13
SPHK2	24.0	3.15	-3.53	0.995	1.92
SPTLC1	22.8	2.01	-3.03	0.997	2.14
SPTLC2	23.0	2.02	-2.99	0.995	2.16
SREBF1	24.1	1.92	-2.96	0.996	2.18
SREBF2	25.4	2.63	-3.60	0.997	1.90
THRSP	28.1	6.87	-3.45	0.998	1.95
VLDLR	23.8	1.58	-3.06	0.994	2.12
UGCG	22.4	2.65	-2.68	0.991	2.36
XDH	18.0	-3.37	-3.27	0.996	2.02

[1] The median is calculated considering all time points and all animals
[2] The median of ΔCt is calculated from [Ct gene – Ct internal control] for each time point and each animal
[3] The R2 stand for the coefficient of determination of the standard curve
[4] The efficiency is determined by $[10^{(-1/Slope)}]$

*

Milk fatty acid composition. Percentage (g/100g) and yield (mole/d) of selected fatty acids in milk fat is reported in Table S6 and 7. Yield of most of the long-chain FA (≥16-carbons) was affected by stage of lactation. Fatty acids >16-carbons decreased over time, and fatty acids with <16-decreased numerically resulting in a change of the ratio between >16-carbon/<16-carbon FA over time. All medium-chain FA (from 12- to 15-carbons) had a similar pattern, with a peak at 120 d. Among short-chain FA, butyrate had the higher yield at 15 d and decreased thereafter. Among FA with 16-carbons, palmitate increased numerically with maximum yield at 30 d.

Palmitoleic acid yield peaked at 15 d and decreased markedly thereafter (Table S7) likely as a consequence of decreased Δ^9 desaturase activity towards 16:0 (Table S6). Data suggest that most milk palmitate was taken up from blood at the beginning of lactation, when blood NEFA rich in palmitate peaked [8, 9]. Subsequently, it appears that most milk palmitate originated from *de novo* synthesis perhaps as a result of greater feed intake and availability of acetate and butyrate [10, 11]. This is supported by the higher yield of stearate, one of the major constituent of NEFA in bovine [9], during the first month of lactation followed by a gradual decrease thereafter (Table 1), and also expressed by the molar proportion of this fatty acid which decreased significantly throughout lactation (Table S6). Greater amount of milk fat *cis*9-16:1 at the beginning of lactation has been reported previously [12]. The quantity of *cis*9-16:1 in blood NEFA is not high [9], suggesting this FA is almost exclusively synthesized via Δ^9 desaturase. In contrast, the greater amount of stearic acid in milk fat and the pattern of oleic acid (larger at 30 vs. 15 d; Table S7) at the beginning of lactation suggest that desaturation of 18:0 at this physiological stage is lower than palmitic acid. This suggestion also is supported by the similar amount of both FA in blood NEFA during the periparturient period in cows [9]. Thus, early post-partum there is greater activity of SCD towards palmitic acid than stearic acid.

*

Table S6. Molar % of FA composition in fat milk during lactation in Holstein dairy cows.

	Day Relative to Parturition						
**Fatty Acid**	**15**	**30**	**60**	**120**	**240**	**SEM**	***P*-Value**
4:0	19.4	18.9	18.5	17.3	15.0	0.77	<0.01
6:0	3.72	4.32	4.66	4.62	4.02	0.23	0.02
8:0	1.31	1.60	1.87	2.04	1.81	0.14	<0.01
10:0	1.95	2.33	3.00	3.67	3.40	0.32	<0.01
11:0	0.06	0.05	0.07	0.11	0.11	0.02	0.02
12:0	1.48	1.74	2.31	3.05	2.96	0.25	<0.01
14:0	5.90	6.72	8.39	9.92	9.81	0.56	<0.01
14:1*cis*9	0.54	0.62	0.81	1.07	1.15	0.09	<0.01
15:0	0.55	0.62	0.76	0.89	0.89	0.06	<0.01
16:0	23.4	23.6	24.1	25.7	25.3	0.65	0.01
16:1*cis*9	2.48	2.01	1.74	1.81	1.88	0.18	0.02
16:1*cis*11	0.048	0.033	0.030	0.025	0.027	0.006	0.001
16:1*trans*9	0.033	0.035	0.036	0.042	0.046	0.005	0.30
16:1*trans*11	0.30	0.28	0.27	0.26	0.30	0.01	0.18
17:0	0.58	0.51	0.45	0.41	0.40	0.02	<0.01
18:0	11.8	10.8	9.7	7.9	9.1	0.67	<0.01
18:1*cis*9	18.8	18.0	15.4	13.4	15.5	0.93	<0.01
18:1*cis*11	0.95	0.83	0.74	0.56	0.52	0.05	<0.01
18:1*cis*12	0.47	0.47	0.52	0.58	0.57	0.03	<0.01
18:1*cis*13	0.11	0.08	0.07	0.05	0.05	0.01	<0.01
18:1*cis*15	0.08	0.07	0.09	0.11	0.12	0.006	<0.01
18:1*trans*4	0.019	0.021	0.020	0.019	0.019	0.001	0.46
18:1*trans*5	0.017	0.013	0.015	0.015	0.015	0.001	0.17
18:1*trans*6,7,8	0.18	0.18	0.19	0.18	0.20	0.01	0.54
18:1*trans*9	0.27	0.27	0.29	0.28	0.31	0.02	0.25
18:1*trans*10	0.27	0.31	0.35	0.36	0.33	0.03	<0.01
18:1*trans*11	0.36	0.33	0.37	0.37	0.47	0.03	<0.01
18:1*trans*12	0.34	0.36	0.41	0.43	0.46	0.02	0.01
18:1*trans*13	0.55	0.56	0.66	0.72	0.72	0.05	0.02
18:1*trans*16	0.27	0.29	0.33	0.31	0.35	0.02	0.02
18:2*cis*9,*cis*12	2.57	2.72	2.48	2.34	2.50	0.12	0.03
18:2*cis*9,*trans*11	0.19	0.22	0.24	0.30	0.37	0.02	<0.01
18:2*cis*9,*trans*12	0.053	0.056	0.056	0.058	0.064	0.002	0.12
18:2*trans*9,*cis*13	0.12	0.14	0.14	0.14	0.13	0.007	0.13
18:2*trans*9,*trans*12	0.021	0.014	0.019	0.025	0.026	0.002	<0.01
Other CLA	0.064	0.056	0.070	0.073	0.097	0.008	0.02

*

Table S6 (cont.). Molar % of FA composition in fat milk during lactation in Holstein dairy cows.

	Day Relative to Parturition						
**Fatty Acid**	**15**	**30**	**60**	**120**	**240**	**SEM**	***P*-Value**
18:*n*3	0.34	0.34	0.29	0.29	0.23	0.01	<0.01
18:3*n*6	0.024	0.023	0.029	0.035	0.039	0.003	<0.01
20:0	0.096	0.095	0.093	0.086	0.101	0.005	<0.01
20:2*n*6	0.031	0.027	0.024	0.024	0.038	0.003	0.03
20:3*n*6	0.11	0.09	0.11	0.14	0.18	0.03	<0.01
20:4*n*6	0.12	0.12	0.11	0.11	0.14	0.01	0.02
20:5*n*3	0.008	0.015	0.019	0.017	0.014	0.002	<0.01
22:0	0.040	0.040	0.038	0.039	0.047	0.003	0.26
22:4*n*6	0.015	0.023	0.046	0.045	0.033	0.014	0.51
22:5*n*3	0.047	0.049	0.039	0.047	0.047	0.005	0.63
24:0	0.015	0.039	0.029	0.056	0.047	0.013	0.36
Δ^9 14:0 to 14:1*c*9	0.089	0.085	0.088	0.097	0.106	0.008	<0.01
Δ^9 16:0 to 16:1*c*9	0.095	0.078	0.067	0.066	0.069	0.007	<0.01
Δ^9 18:0 to 18:1*c*9	0.61	0.63	0.61	0.63	0.63	0.02	0.77
Δ^9 18:1*t*11 to 18:2*c*9,*t*11	0.33	0.40	0.40	0.44	0.44	0.06	0.01
Δ^6 18:2*c*9,*c*12 to 18:3*n*6	0.009	0.009	0.012	0.014	0.015	0.001	<0.01
Δ^5 20:3*n*6 to 20:4*n*6	0.56	0.61	0.54	0.48	0.47	0.06	<0.01
<16C	34.9	36.9	40.3	42.6	39.3	1.34	<0.01
16C (16:0 + 16:1)	26.3	26.0	26.2	27.8	27.6	0.75	0.09
>16C	38.8	37.1	33.5	29.5	33.2	1.37	<0.01
<16C/>16C	0.91	1.00	1.21	1.48	1.20	0.09	<0.01
Unsaturated	70.2	71.4	74.0	75.8	73.1	1.10	<0.01
Saturated	29.8	28.6	26.0	24.2	26.9	1.10	<0.01
Synthesized	46.4	48.9	53.5	57.8	52.8	2.08	<0.01
Imported	53.6	51.1	46.5	42.2	47.2	2.08	<0.01
ACE mol/mol[1]	1.74	1.82	1.99	2.22	2.17	0.76	<0.01
ACE mol/mol*	1.70	1.78	1.94	2.16	2.11	0.73	<0.01
ACE mol/d[1]	11.4	14.0	13.8	12.9	12.1	1.54	0.46
ACE mol/d*	11.2	13.7	13.5	12.6	11.7	1.50	0.45
ACEc mol/d[2]	5.2	14.0	13.8	12.9	12.0	1.46	<0.01
ACEc mol/d*	4.9	13.7	13.5	12.6	11.7	1.42	<0.01

[1] Calculated as (chain length/2 – 1.5) for FA 4:0 to 14:1 and (chain length/2 – 1.5) × 0.6 for 16:0 and 16:1 (see [12])

[2] ACE corrected for fat at 15 d and calculated as (chain length/2 – 1.75) for FA 4:0 to 14:1 and (chain length/2 – 1.75) × 0.1 for 16:0 and 16:1 to account for large up-take of FA from NEFA blood during the first two week of lactation ([12]).

*Exclusion of odd chain FA from ACE calculation considering those FA deriving exclusively from rumen bacteria (e.g. taken up from plasma).

*

Table S7. Yield (mole/d) of fatty acid composition of fat milk throughout the entire lactation.

	Day Relative to Parturition						
**Fatty Acid**	**15**	**30**	**60**	**120**	**240**	**SEM**	***P*-Value**
4:0	1260	1488	1275	973	833	168	<0.01
6:0	249.9	334.6	318.5	266.4	222.7	40	0.06
8:0	90.0	122.5	129.6	119.2	100.1	18	0.20
10:0	136.0	176.4	208.2	217.0	189.5	34	0.19
11:0	4.0	3.6	5.3	6.5	6.5	1.5	0.18
12:0	102.7	131.5	160.6	180.9	166.1	27	0.07
14:0	397.1	511.7	583.2	583.2	543.9	78	0.16
14:1*cis*9	36.7	47.1	56.5	63.0	65.2	8.9	0.03
15:0	37.3	46.9	53.2	51.9	49.9	6.6	0.18
16:0	1515	1834	1669	1488	1403	198	0.23
16:1*cis*9	158.3	156.2	120.8	103.7	104.2	2.0	0.03
16:1*cis*11	3.2	2.5	2.1	1.4	1.6	0.4	<0.01
16:1*trans*9	2.1	2.9	2.4	2.3	2.6	0.5	0.66
16:1*trans*11	19.2	22.0	18.2	14.4	16.1	2.4	0.04
17:0	37.0	39.8	31.0	22.9	22.1	4.1	<0.01
18:0	755.2	854.3	659.4	432.7	486.6	99.8	<0.01
18:1*cis*9	1207	1410	1059	743	848	152	<0.01
18:1*cis*11	62.4	63.5	51.2	31.3	28.9	6.4	<0.01
18:1*cis*12	30.1	37.0	35.7	33.0	31.4	4.6	0.54
18:1*cis*13	7.3	6.1	4.6	2.6	2.6	1.0	<0.01
18:1*cis*15	5.1	5.7	6.5	6.3	6.4	0.9	0.48
18:1*trans*4	1.2	1.7	1.4	1.1	1.1	0.2	0.03
18:1*trans*5	1.1	1.0	1.1	0.8	0.8	0.2	0.28
18:1*trans*6,7,8	11.7	14.1	12.7	10.3	11.2	1.8	0.24
18:1*trans*9	16.7	20.7	19.8	15.7	17.0	2.6	0.27
18:1*trans*10	17.6	23.6	23.8	20.3	18.2	2.9	0.13
18:1*trans*11	23.4	26.0	25.2	20.9	25.4	4.0	0.67
18:1*trans*12	21.9	28.4	27.8	24.3	25.4	3.9	0.46
18:1*trans*13	35.7	44.0	44.9	41.0	40.3	6.8	0.69
18:1*trans*16	17.7	22.9	22.2	17.6	19.4	3.1	0.28
18:2*cis*9,*cis*12	164.3	216.1	168.3	131.1	136.7	24.6	0.02
18:2*cis*9,*trans*11	11.9	16.9	16.8	17.2	20.5	2.5	0.07
18:2*cis*9,*trans*12	3.5	4.4	3.8	3.3	3.5	0.6	0.38
18:2*trans*9,*cis*12	8.2	11.5	9.6	8.1	7.2	1.3	0.04
18:2*trans*9,*trans*12	1.4	1.1	1.3	1.4	1.5	0.3	0.69
Other CLA	4.2	4.3	4.8	4.2	5.5	0.9	0.59

*

Table S7 (cont.). Yield (mole/d) of fatty acid composition of fat milk throughout the entire lactation.

	Day Relative to Parturition						
**Fatty Acid**	**15**	**30**	**60**	**120**	**240**	**SEM**	***P*-Value**
18:*n*3	22.0	26.5	20.0	16.1	12.7	2.9	<0.01
18:3*n*6	1.6	1.9	2.0	1.9	2.2	0.3	0.46
20:0	6.2	7.4	6.4	4.8	5.5	0.8	0.03
20:2*n*6	2.1	2.1	1.6	1.4	2.0	3.6	0.18
20:3*n*6	7.3	6.8	7.5	7.6	10.0	1.4	0.26
20:4*n*6	7.9	9.4	7.6	6.5	8.1	1.0	0.08
20:5*n*3	0.5	1.2	1.3	1.0	0.7	0.3	<0.01
22:0	2.7	3.1	2.6	2.2	2.5	4.0	0.25
22:4*n*6	1.2	1.6	3.1	2.8	1.9	1.3	0.54
22:5*n*3	3.2	3.7	2.6	2.7	2.6	0.6	0.23
24:0	0.9	2.9	1.8	3.1	2.7	1.2	0.39
<16C	2309	2863	2791	2461	2188	346	0.25
>16C	2505	2919	2287	1640	1804	304	<0.01
16C[1]	1698	2018	1813	1610	1528	217	0.21
<16/>16	0.91	1.00	1.21	1.48	1.20	0.11	<0.01
Saturate	1920	2243	1786	1359	1480	234	<0.01
Unsaturate	4592	5557	5105	4352	4056	609	0.13
Unsaturate/Saturate	2.4	2.5	2.9	3.2	2.8	0.2	<0.01

[1] Sum of 16:0 + 16:1 (all geometrical isomers)

*

Figure S2. Delta 5 and delta 6 desaturase indexes during lactation in Holstein dairy cows. The Δ^5 was calculated as [20:4n6/(20:4n6 + 20:3n6)] (SEM = 0.06) and Δ^6 as [18:3n6/(18:3n6 + 18:2*cis*9,*cis*12)] (SEM = 0.001). Statistical effect of time: $P < 0.05$ for both indexes.

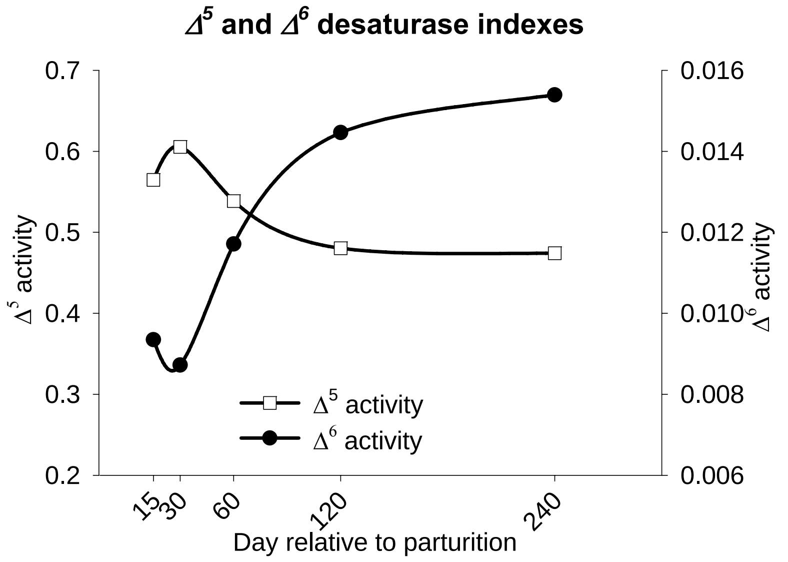

*

Figure S3. Possible utilization of β-hydroxybutyrate in bovine mammary gland. The figure was generated with qPCR data from the present manuscript and microarray data from a large longitudinal bovine mammary gene expression analysis [13]. The pathway was built based on Robinson and Williamson [14] with modifications. On the cytosol, BHBA is incorporated as a 4-unit carbon molecule directly into FA by acetyl-CoA carboxylase (*ACACA*). Before incorporation into FA, BHBA has to be reduced and activated to butyril-CoA. We did not measure mRNA of any of those enzymes by qPCR, and the bovine microarray platform does not contain sequences for all genes in this pathway (a gene sequence for a cytosolic enoyl-CoA reductase is not even available for mammals). Bovine have very low amounts of butyrate in blood [15], thus, the direct activation of butyrate to butyril-CoA probably is a negligible reaction in mammary tissue.

Our data suggest that a large portion of BHBA enters the mitochondria to replenish the TCA cycle but it is not necessarily utilized as an energy source as indicated by the low level of $^{14}CO_2$ after incubation with labeled BHBA [16]. The major fate of BHBA in bovine mammary tissue is the synthesis of citrate. Citrate is readily secreted in milk [12] and it can potentially furnish NADPH in the cytosol (via citrate/α-ketoglutarate shunt). However, inverse relationships between acetoacetate and lipogenesis [14] as well as citrate and *de novo* synthesis of FA [12] have been reported. The mRNA abundance of the major part of enzymes required for utilization of citrate in the TCA cycle, for production of NADPH, or, indirectly, for furnishing precursors for amino acids tended to decrease during lactation. In addition, our data suggest scarce utilization of acetyl-CoA resulting from cleavage of BHBA for cytosolic FA synthesis. The inability of bovine mammary tissue to utilize acetyl-CoA generated in mitochondria to synthesize FA in cytosol is well documented [17]. However, utilization of acetyl-CoA from BHBA to generate FA in mammary tissue mitochondria has been demonstrated[18]. The ability of mitochondria to synthesize FA has become more evident in recent studies (e.g.[19]). Our microarray data [13] revealed up-regulation of the first two steps of the mitochondrial FA synthesis pathway during lactation (see Figure S4).

The SLC16A family of transporters contains many isoforms with similar affinity for ketone bodies, lactate, and pyruvate [20]. In our microarray data, the transcript of *SLC16A7* (with high affinity for BHBA) was up-regulated during lactation. The SLC16A1 isoform has greater affinity for acetoacetate, which can result in provision of acetoacetate from the mitochondria to the cytosol. As observed by qPCR data, mRNA of the two enzymes involved in the first steps of the utilization of BHBA in the mitochondria were largely up-regulated during lactation, suggesting substantial entry of BHBA into the mithocondria (see main body of the article). Those data are in agreement with previously reported activity of the same enzyme in rat mammary tissue during lactation [21]. Strangely enough, thiolase (*ACAT1*) expression tended to be down-regulated during lactation, which does not agree with activity of this enzyme in lactating rat mammary tissue [21]. The utilization of acetoacetate produced by BDH1 for cytosolic FA synthesis is not supported by our gene expression data. In fact, mRNA abundance of the two major enzymes for the cleavage of this compound into acetyl-CoA (acetoacetyl-CoA synthetase – *AACS* – and acetyl-CoA acetyltransferase 2 – *ACAT2*) was down-regulated. Among the enzymes involved in the TCA cycle we observed transcript up-regulation of citrate synthetase (*CS*) and aconitase (*ACO2*). Those data

*

seem to suggest that the utilization of acetyl-CoA cleaved from BHBA for citrate synthesis is pivotal. Expression of the other TCA cycle-related genes was down-regulated or remained flat during lactation. An exception was the succinate dehydrogenase complex, subunit D (*SDHD*), an integral membrane protein linking oxidative phosphorilation to the TCA. Transcripts of the genes involved in the utilization of TCA intermediates for amino acid synthesis were down-regulated (e.g. *GOT2*). Citrate needs to be present in the cytosol to be excreted in milk. The down-regulation in expression of the specific citrate transporter (*SLC25A1*) suggests that there exist other means for transportation of citrate from mitochondria into the cytosol. We also observed mRNA up-regulation of carnitine acetyltransferase (*CRAT*). The movement of acetyl-CoA from mitochondria to the cytosol by this transporter has been extensively investigated [17]. Research has clearly shown that this transporter is present only in the inner side of mitochondrial membrane, but there does not appear to be an external acceptor for carnitine. Among all the acyl-carnitine-related transcripts in the bovine microarray platform, the expression of *CRAT* had the largest up-regulation during lactation, suggesting a specific function of the protein product in "managing" the fate of mitochondrial acetyl-CoA during lactation.

*

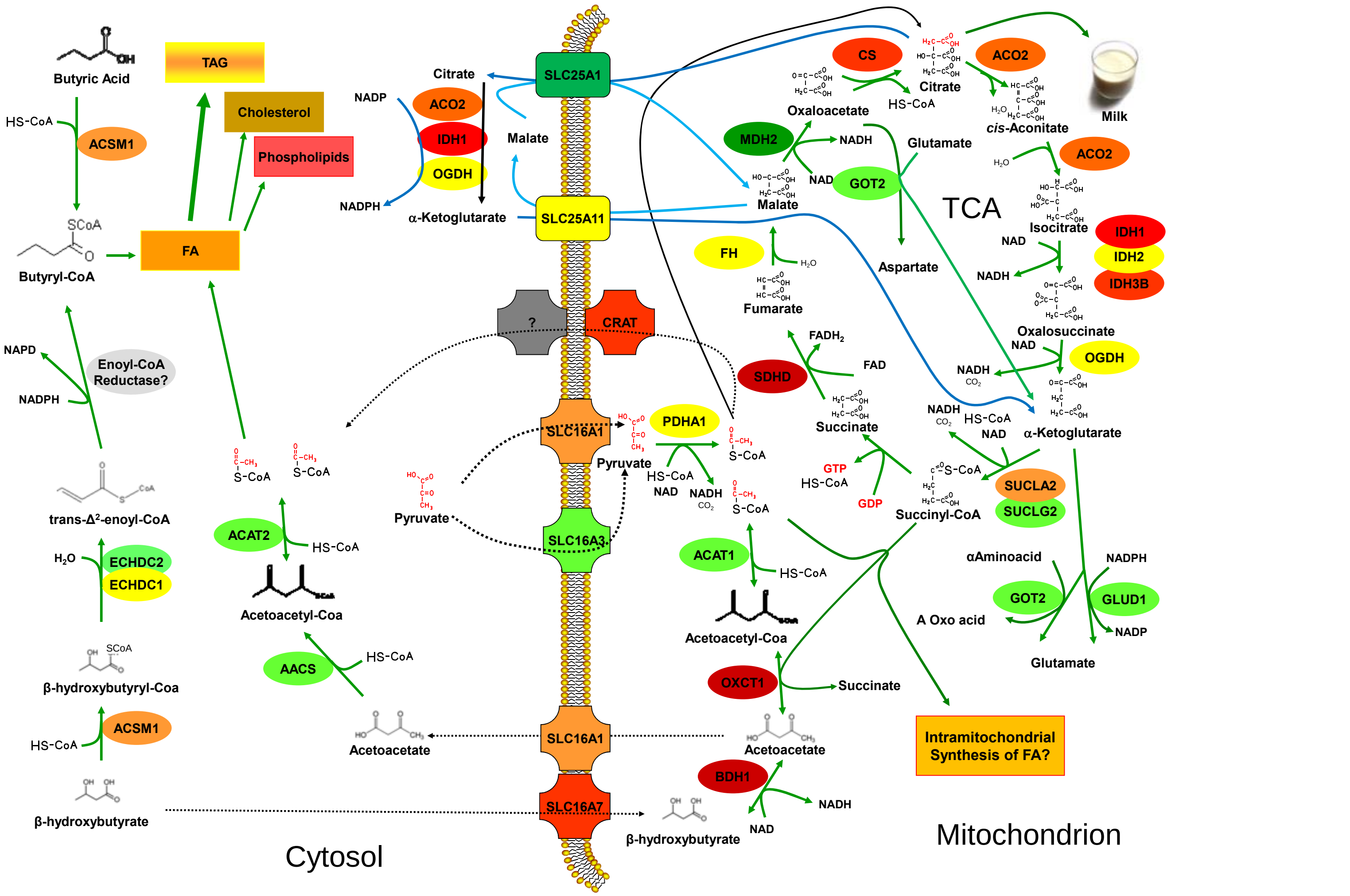

The round shaped objects denote enzymes, big rectangles denote main products (e.g. TAG = tryacylglycerol), other shapes denote transporters. Within the objects are reported the accepted symbol of the gene coding for the corresponding proteins (NCBI). The colors of the shapes denote: ● = >5-fold; ● = >2-fold; ● = 1.2÷2-fold ●= 1÷1.2-fold; ● = -1÷2-fold; ● = <-2-fold between 15 and 120 d of lactation vs. -15/-30 d (or pregnancy). All array data had overall time effect with a false discovery rate < 0.05 [13].

*

Figure S4. **Possible pathway for the mithocondrial FA synthesis** In 1972 McCarthy and Smith [18] were able to demonstrate direct formation of FA in mitochondria of bovine mammary cells. They suggested the presence of a fatty acid synthetase complex in mithocondria based on those data. This hypothesis was not taken into consideration until very recently, when several discoveries clearly showed that mammalian mitochondria are able to synthesized FA because they contain several enzymes possessing high similarity to bacterial fatty acid synthesis enzymes (e.g. [22-24]). Early work also demonstrated the capacity of bovine mitochondria to elongate fatty acids by insertion of acetyl-CoA [19]. Based on the original data from McCarthy and Smith [18], this pathway appears to have little importance in the utilization of BHBA for FA synthesis. However, it was intriguing that the expression of malonyl CoA:ACP acyltransferase (mitochondrial) (*MCAT*) and 3-oxoacyl-ACP synthase, mitochondrial (*OXSM*), the only enzymes involved in the mithocondrial FA synthesis with a mammalian mRNA sequence available, was up-regulated during lactation. This is suggestive of a functional role of this pathway in the synthesis of mitochondrial FA. Other enzymes in this pathway have not been defined clearly in mammals. Thus, wer incorporated the bacterial enzyme names into the pathway (as reported in Bhaumik et al.[24]).

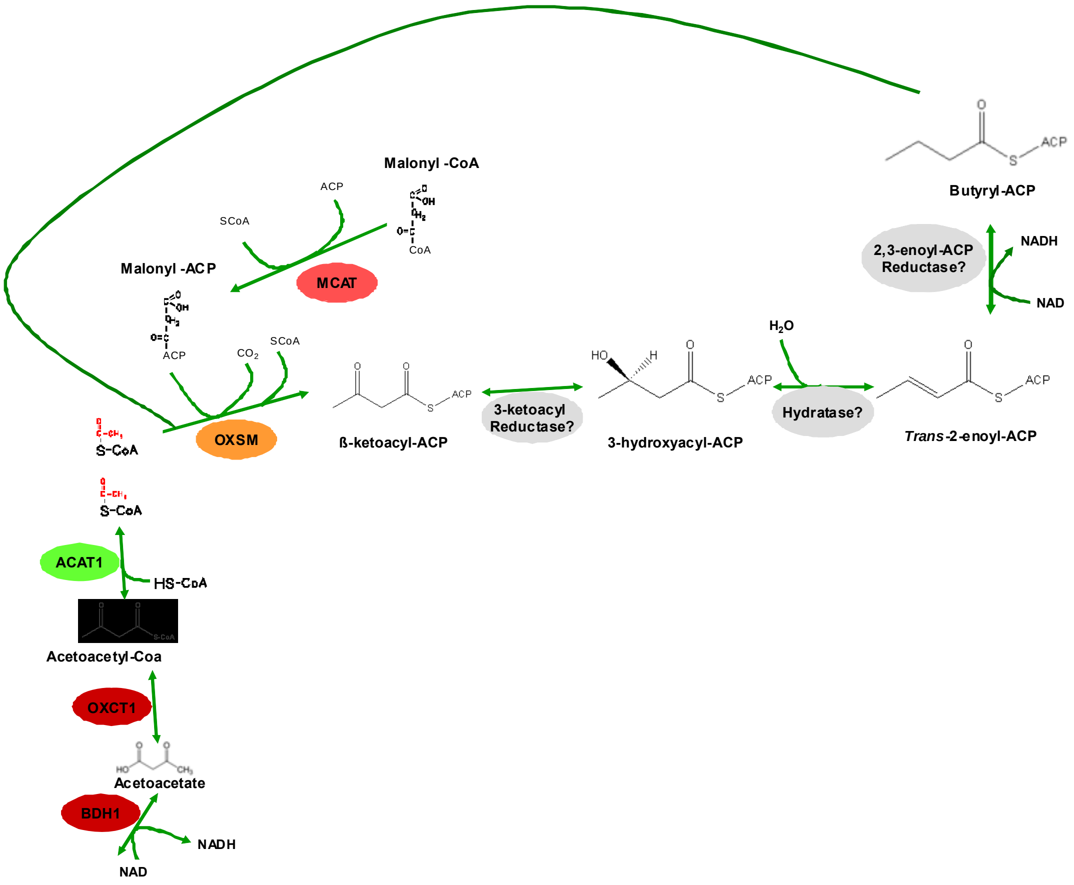

*

Figure S5. **Implication of enzymes involved in sphingolipid synthesis**. The pathway is focused on ceramide synthesis, including subcellular location of enzymes. The figure was built using information from several manuscripts [25-27]. Enzymes with measured temporal mRNA are colored (red = up-regulated; green = down-regulated; and yellow = no change during lactation vs. dry period or -15 d). Red arrows denote synthesis of ceramide; dark green arrows degradation of ceramide; orange arrow denotes synthesis of sphingolipid-1-P; light green arrows denote degradation of sphingolipid-1-P.

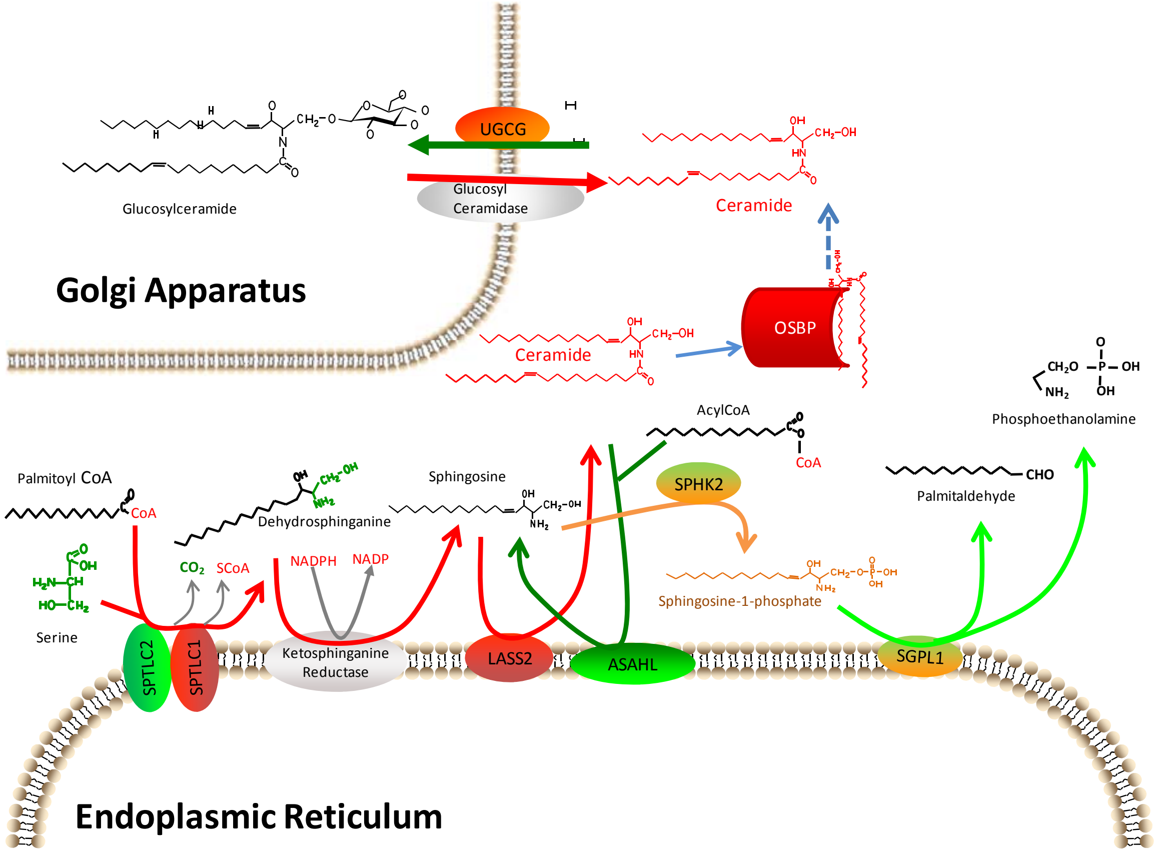

*

Supplemental discussion for Figure 6 in main body.

Among the 45 genes tested, IPA uncovered a single large network encompassing 32 genes. At the center of this network were *SREBF1*, *SREBF2*, and *PPARG*. Published literature has shown that *SREBF1* positively induces expression of 11 down-stream genes: *LPL* and *DBI* (involved in FA uptake), all 3 desaturases (*SCD*, *FADS1*, and *FADS2*), 3 key genes associated with the FA and TAG synthesis machinery (*ACACA*, *FASN*, and *GPAM*), acyl-CoA synthetases (*ACSS2* and *ACSL1*), the nuclear receptor *THRSP*. Previous works also have shown that the protein encoded by *SREBF2* induces gene expression of most genes regulated by *SREBF1*, with addition of *INSIG1*. Expression and function of the SREBP1 and 2 are under control of *SCAP* and *INSIG1,* whereas *INSIG2* regulates the expression of *SREBF1* only.

The protein encoded by *PPARG* plays a central role in the induction of 9 genes measured (*ACSL1*, *SCD*, *ACACA*, *CD36*, *ADFP*, *ABCA1*, *PLIN*, *DGAT1*, and *LPL*). *ABCG2* has a protein-DNA interaction with *PPARG* due to the presence of a PPRE on its promoter region. *PPARGC1A* induces expression of *LPIN1,* which indirectly induces expression of other genes (*SCD*, *ACACA*, *FASN*, and *DGAT1*) through the formation of the complex (protein-protein interaction) with *PPARG*. There were no additional interactions in the IPA database for remaining genes, but a large literature search revealed that *OSBP* directly regulates expression of *SREBF1* [28], whereas *SPHK2* activity indirectly affects SREBP activation because it degrades ceramides, known activators of SREBF1 [29]. Expression of specific *PPARG* agonists increases activity of *SPTLC1* and *ASHAL* [30]. Those relationships were not present in the original IPA analysis and were added manually. Other relationships, such as protein-protein interactions for *ACSL1*, *FABP3*, and *CD36* where added based on our results and previous findings, as discussed in the main body of the paper. Other genes such as *ACSS1*, *AGPAT6*, *BDH1*, *OXCT1*, *SGPL1*, *LASS2*, *OSBPL10*, *OSBPL2*, and *UCGC* have no apparent relationships in the IPA database and where not added in the networks.

*

References for Supplementary Materials

1. Loor JJ, Dann HM, Guretzky NA, Everts RE, Oliveira R, Green CA, Litherland NB, Rodriguez-Zas SL, Lewin HA, Drackley JK: **Plane of nutrition prepartum alters hepatic gene expression and function in dairy cows as assessed by longitudinal transcript and metabolic profiling.** *Physiol Genomics* 2006, **27:**29-41.
2. Bionaz M, Loor JJ: **Identification of reference genes for quantitative real-time PCR in the bovine mammary gland during the lactation cycle.** *Physiol Genomics* 2007, **29:**312-319.
3. Loor JJ, Dann HM, Everts RE, Oliveira R, Green CA, Guretzky NA, Rodriguez-Zas SL, Lewin HA, Drackley JK: **Temporal gene expression profiling of liver from periparturient dairy cows reveals complex adaptive mechanisms in hepatic function.** *Physiol Genomics* 2005, **23:**217-226.
4. Bionaz M, Loor JJ: **ACSL1, AGPAT6, FABP3, LPIN1, and SLC27A6 are the most abundant isoforms in bovine mammary tissue and their expression is affected by stage of lactation.** *J Nutr* 2008, **138:**1019-1024.
5. Harvatine KJ, Bauman DE: **SREBP1 and thyroid hormone responsive spot 14 (S14) are involved in the regulation of bovine mammary lipid synthesis during diet-induced milk fat depression and treatment with CLA.** *J Nutr* 2006, **136:**2468-2474.
6. Farr VC, Stelwagen K, Cate LR, Molenaar AJ, McFadden TB, Davis SR: **An improved method for the routine biopsy of bovine mammary tissue.** *J Dairy Sci* 1996, **79:**543-549.
7. Loor JJ, Herbein JH: **Reduced fatty acid synthesis and desaturation due to exogenous trans10,cis12-CLA in cows fed oleic or linoleic oil.** *J Dairy Sci* 2003, **86:**1354-1369.
8. Grum DE, Drackley JK, Younker RS, LaCount DW, Veenhuizen JJ: **Nutrition during the dry period and hepatic lipid metabolism of periparturient dairy cows.** *J Dairy Sci* 1996, **79:**1850-1864.
9. Leroy JL, Vanholder T, Mateusen B, Christophe A, Opsomer G, de Kruif A, Genicot G, Van Soom A: **Non-esterified fatty acids in follicular fluid of dairy cows and their effect on developmental capacity of bovine oocytes in vitro.** *Reproduction* 2005, **130:**485-495.
10. Schwalm JW, Schultz LH: **Relationship of insulin concentration to blood metabolites in the dairy cow.** *J Dairy Sci* 1976, **59:**255-261.
11. Storry JE, Rook JA: **Effects of intravenous infusions of acetate, beta-hydroxybutyrate, triglyceride and other metabolites on the composition of the milk fat and blood in cows.** *Biochem J* 1965, **97:**879-886.
12. Garnsworthy PC, Masson LL, Lock AL, Mottram TT: **Variation of milk citrate with stage of lactation and de novo fatty acid synthesis in dairy cows.** *J Dairy Sci* 2006, **89:**1604-1612.
13. Bionaz M, Rodriguez-Zas SL, Everts RE, Lewin HA, Loor JJ: **MammOmics™: transcript profiling of the mammary gland during the lactation cycle in Holstein cows.** In *ADSA-PSA-ASAS-AMPA Joint Annual Meeting*. J Dairy Sci; 2007: (Suppl1):272.

*

14. Robinson AM, Williamson DH: **Physiological roles of ketone bodies as substrates and signals in mammalian tissues.** *Physiol Rev* 1980, **60:**143-187.
15. Black AL, Kleiber M, Brown AM: **Butyrate Metabolism in the Lactating Cow.** *J Biol Chem* 1961, **236:**2399-2403.
16. Palmquist DL, Davis CL, Brown RE, Sachan DS: **Availability and Metabolism of Various Substrates in Ruminants. V. Entry Rate into the Body and Incorporation into Milk Fat of D( ){beta}-Hydroxybutyrate.** *J Dairy Sci* 1969, **52:**633-638.
17. Bauman DE, Davis CL: **Biosynthesis of milk fat.** In *Lactation: a comprehensive treatise. Volume* 2. Edited by Larson BL, Smith VR. New York
London: Academic Press; 1974: 31-75
18. McCarthy S, Smith GH: **Synthesis of milk fat from -hydroxybutyrate and acetate by ruminant mammary tissue in vitro.** *Biochim Biophys Acta* 1972, **260:**185-196.
19. Witkowski A, Joshi AK, Smith S: **Coupling of the de novo fatty acid biosynthesis and lipoylation pathways in mammalian mitochondria.** *J Biol Chem* 2007, **282:**14178-14185.
20. Halestrap AP, Meredith D: **The SLC16 gene family-from monocarboxylate transporters (MCTs) to aromatic amino acid transporters and beyond.** *Pflugers Arch* 2004, **447:**619-628.
21. Page MA, Williamson DH: **Lactating mammary gland of the rat: a potential major site of ketone-body utilization.** *Biochem J* 1972, **128:**459-460.
22. Hiltunen JK, Okubo F, Kursu VA, Autio KJ, Kastaniotis AJ: **Mitochondrial fatty acid synthesis and maintenance of respiratory competent mitochondria in yeast.** *Biochem Soc Trans* 2005, **33:**1162-1165.
23. Cronan JE, Fearnley IM, Walker JE: **Mammalian mitochondria contain a soluble acyl carrier protein.** *FEBS Lett* 2005, **579:**4892-4896.
24. Bhaumik P, Koski MK, Glumoff T, Hiltunen JK, Wierenga RK: **Structural biology of the thioester-dependent degradation and synthesis of fatty acids.** *Curr Opin Struct Biol* 2005, **15:**621-628.
25. Futerman AH, Riezman H: **The ins and outs of sphingolipid synthesis.** *Trends Cell Biol* 2005, **15:**312-318.
26. Tani M, Ito M, Igarashi Y: **Ceramide/sphingosine/sphingosine 1-phosphate metabolism on the cell surface and in the extracellular space.** *Cell Signal* 2007, **19:**229-237.
27. Reiss U, Oskouian B, Zhou J, Gupta V, Sooriyakumaran P, Kelly S, Wang E, Merrill AH, Jr., Saba JD: **Sphingosine-phosphate lyase enhances stress-induced ceramide generation and apoptosis.** *J Biol Chem* 2004, **279:**1281-1290.
28. Yan D, Lehto M, Rasilainen L, Metso J, Ehnholm C, Yla-Herttuala S, Jauhiainen M, Olkkonen VM: **Oxysterol binding protein induces upregulation of SREBP-1c and enhances hepatic lipogenesis.** *Arterioscler Thromb Vasc Biol* 2007, **27:**1108-1114.
29. Worgall TS, Juliano RA, Seo T, Deckelbaum RJ: **Ceramide synthesis correlates with the posttranscriptional regulation of the sterol-regulatory element-binding protein.** *Arterioscler Thromb Vasc Biol* 2004, **24:**943-948.

*

30. Baranowski M, Blachnio A, Zabielski P, Gorski J: **Pioglitazone induces de novo ceramide synthesis in the rat heart.** *Prostaglandins Other Lipid Mediat* 2007, **83:**99-111.

*
